# Supplementary material for: Individual differences in alexithymia modulate cognition-emotion interactions in daily life ongoing experiences
Source: Commun Psychol. 2026 Mar 10;4:71. doi: 10.1038/s44271-026-00434-7 (PMC13106792; doi:10.1038/s44271-026-00434-7)
Supplement: Supplementary file 2 — Supplementary Information [file 44271_2026_434_MOESM2_ESM.pdf]

# Supplementary Materials

**Supplementary Table 1.** A summary of alexithymia-related measures and baseline distress for participants in the current study ($N = 190$). TAS: Toronto-Alexithymia Scale. DIF: Difficulty identifying feelings; DDF: Difficulty describing feelings; EOT: Externally oriented thinking; DASS: Depression, Anxiety, and Stress Scale.

| | Mean score (*SD*) | Range |
|---|---|---|
| TAS-20 total | *M* = 55.5 (10.0) | 32-80 |
| TAS-DIF | *M* = 19.9 (5.8) | 7-35 |
| TAS-DDF | *M* = 15.9 (4.2) | 6-24 |
| TAS-EOT | *M* = 19.8 (3.6) | 10-37 |
| DASS-21 total | *M* = 47.35 (26.72) | 0-124 |

**Supplementary Table 2.** Descriptive statistics of experience-sampling measures by alexithymia level. Mean response time (seconds), number of responses, and indices of within-person and within-assessment variability for thought ratings and affect ratings, reported separately for participants with low and high scores on TAS-20 total and its subscales (DIF, DDF, EOT). Values are shown as mean (with *SD*). Low and high groups were defined using a median split on each alexithymia measure. *Note*: Within-person variability indexes the within-person standard deviation across repeated assessments, reflecting the breadth of response use over time. Within-assessment variability reflects the standard deviation across items within a single assessment, indexing response dispersion at each sampling point.

| | Mean response time in seconds | Mean number of responses | Mean within-person variability – thought | Mean within-person variability – affect | Mean within-assessment variability – thought | Mean within-assessment variability – affect |
|---|---|---|---|---|---|---|
| TAS-20 total | | | | | | |
| *Low* | 92.12 (22.10) | 26.73 (4.78) | 2.30 (0.54) | 1.90 (0.53) | 2.52 (0.64) | 2.48 (0.70) |
| *High* | 84.61 (20.14) | 26.57 (4.88) | 2.29 (0.42) | 1.99 (0.49) | 2.41 (0.58) | 2.30 (0.62) |
| TAS-DIF | | | | | | |
| *Low* | 90.57 (22.29) | 26.64 (4.77) | 2.28 (0.53) | 1.89 (0.51) | 2.49 (0.65) | 2.45 (0.72) |
| *High* | 85.70 (20.14) | 26.65 (4.91) | 2.31 (0.60) | 2.00 (0.50) | 2.43 (0.55) | 2.31 (0.60) |
| TAS-DDF | | | | | | |
| *Low* | 89.40 (20.84) | 26.88 (4.78) | 2.32 (0.54) | 1.93 (0.55) | 2.51 (0.66) | 2.44 (0.72) |
| *High* | 87.18 (22.13) | 26.38 (4.88) | 2.27 (0.41) | 1.96 (0.46) | 2.41 (0.54) | 2.33 (0.60) |
| TAS-EOT | | | | | | |
| *Low* | 92.25 (22.62) | 26.04 (4.72) | 2.35 (0.48) | 1.99 (0.50) | 2.52 (0.60) | 2.39 (0.65) |
| *High* | 82.90 (18.40) | 27.51 (4.85) | 2.21 (0.48) | 1.88 (0.51) | 2.39 (0.62) | 2.39 (0.69) |

**Supplementary Table 3.** Description of each MDES thought item.

| **Dimensions** | **Questions** | ***0*** | ***10*** |
|---|---|---|---|
| Task | My thoughts were focused on an external task or activity | Not at all | Completely |
| Future | My thoughts involved future events | Not at all | Completely |
| Past | My thoughts involved past events | Not at all | Completely |
| Self | My thoughts involved myself | Not at all | Completely |
| People | My thoughts involved other people | Not at all | Completely |
| Emotion | The emotion of my thoughts was positive (0 = negative) | Negative | Positive |
| External | My thoughts were linked to information from the external environment/surroundings | Not at all | Completely |
| Images | My thoughts involved images | Not at all | Completely |
| Words | My thoughts involved words | Not at all | Completely |
| Sounds | My thoughts involved sounds | Not at all | Completely |
| Detailed | My thoughts were detailed and specific | Not at all | Completely |
| Deliberate | My thoughts were deliberate (0 = spontaneous) | Spontaneous | Deliberate |
| Solutions | I was thinking about solutions to problems (or goals) | Not at all | Completely |
| Intrusive | My thoughts were intrusive | Not at all | Completely |
| Knowledge | My thoughts contained information I already knew (e.g., knowledge or memories) | Not at all | Completely |
| Absorption | I was absorbed in the contents of my thoughts | Not at all | Completely |
| Distracting | My thoughts were distracting me from what I am doing | Not at all | Completely |
| Meaningful | My thoughts were meaningful | Shallow | Meaningful |

**Supplementary Table 4**. Scree plot displaying eigenvalues of principal components generated from the PCA on the experience sampling data.

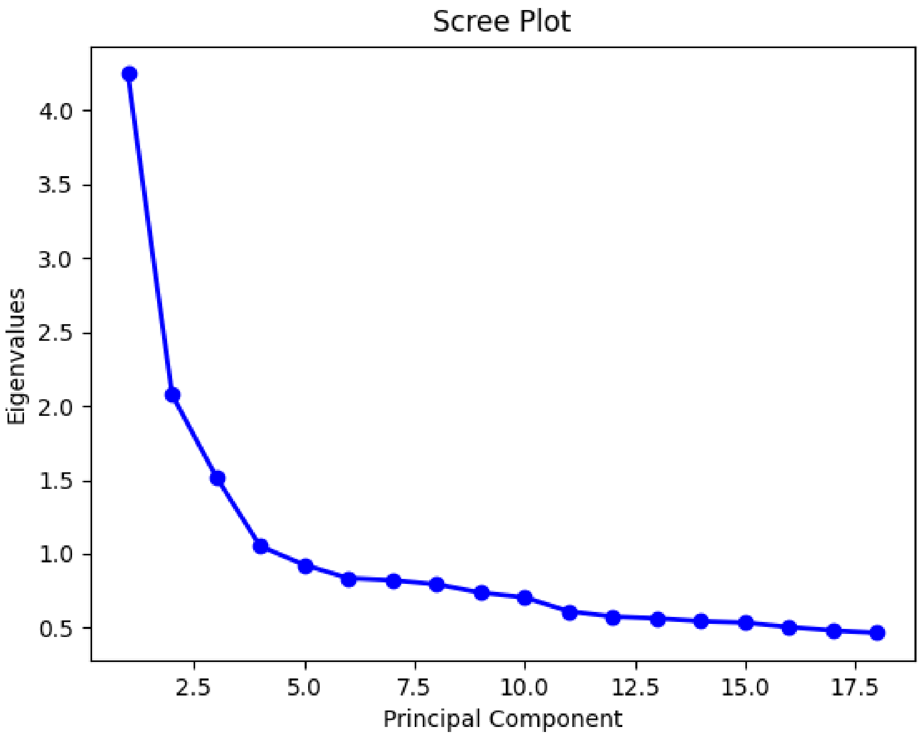

**Supplementary Table 5**. Thought item loadings for each component generated by PCA.

| **Dimension** | **Future-self (Component 1)** | **Intrusive distraction (Component 2)** | **Sensory engagement (Component 3)** | **Task-focus (Component 4)** |
|---|---|---|---|---|
| Task | -0.021 | -0.056 | -0.052 | 0.582 |
| Future | 0.435 | -0.003 | -0.068 | -0.076 |
| Past | 0.251 | 0.212 | 0.135 | -0.226 |
| Self | 0.382 | 0.045 | -0.007 | -0.092 |
| People | 0.210 | -0.063 | 0.343 | -0.157 |
| Emotion | 0.126 | -0.464 | 0.289 | 0.025 |
| External | -0.093 | -0.021 | 0.091 | 0.539 |
| Images | -0.079 | 0.103 | 0.484 | 0.086 |
| Words | 0.131 | -0.031 | 0.349 | -0.018 |
| Sounds | -0.169 | 0.050 | 0.587 | 0.072 |
| Detailed | 0.260 | 0.093 | 0.061 | 0.240 |
| Deliberate | 0.294 | -0.160 | -0.071 | 0.255 |
| Solution | 0.311 | 0.163 | -0.179 | 0.188 |
| Intrusive | -0.019 | 0.521 | 0.084 | -0.057 |
| Knowledge | 0.313 | -0.038 | 0.108 | -0.011 |
| Absorption | 0.076 | 0.318 | 0.048 | 0.320 |
| Distracting | 0.023 | 0.527 | 0.033 | 0.007 |
| Meaningful | 0.355 | -0.061 | 0.038 | 0.060 |

**Supplementary Table 6.** ANOVA outputs and LMM summary tables for the main and interaction effects of affective states (valence, arousal, and stress) on 4 thought dimensions

1: Future-self

| *Row* | *Sum Sq* | *Mean Sq* | *NumDF* | *DenDF* | *F value* | *Pr(>F)* |
|---|---|---|---|---|---|---|
| Valence | 62.571 | 62.571 | 1 | 5053.772 | 107.006 | 0.000 |
| Arousal | 301.300 | 301.300 | 1 | 5048.779 | 515.266 | 0.000 |
| Stress | 169.353 | 169.353 | 1 | 5052.044 | 289.617 | 0.000 |
| Environment | 2.484 | 2.484 | 1 | 5043.992 | 4.249 | 0.039 |
| Age | 12.741 | 12.741 | 1 | 187.524 | 21.789 | 0.000 |
| Gender | 0.723 | 0.361 | 2 | 184.584 | 0.618 | 0.540 |
| Valence:Arousal | 0.459 | 0.459 | 1 | 5047.472 | 0.785 | 0.376 |

2: Intrusive distraction

| *Row* | *Sum Sq* | *Mean Sq* | *NumDF* | *DenDF* | *F value* | *Pr(>F)* |
|---|---|---|---|---|---|---|
| Valence | 162.731 | 162.731 | 1 | 5046.818 | 299.519 | 0.000 |
| Arousal | 79.762 | 79.762 | 1 | 5052.535 | 146.808 | 0.000 |
| Stress | 131.079 | 131.079 | 1 | 5050.106 | 241.261 | 0.000 |
| Environment | 17.331 | 17.331 | 1 | 5023.229 | 31.899 | 0.000 |

| | | | | | | |
|---|---|---|---|---|---|---|
| Age | 0.581 | 0.581 | 1 | 184.028 | 1.070 | 0.302 |
| Gender | 2.761 | 1.380 | 2 | 181.768 | 2.541 | 0.082 |
| Valence:Arousal | 23.342 | 23.342 | 1 | 5028.233 | 42.964 | 0.000 |

3: Sensory engagement

| *Row* | *Sum Sq* | *Mean Sq* | *NumDF* | *DenDF* | *F value* | *Pr(>F)* |
|---|---|---|---|---|---|---|
| Valence | 112.473 | 112.473 | 1 | 5016.238 | 189.853 | 0.000 |
| Arousal | 99.390 | 99.390 | 1 | 5027.862 | 167.769 | 0.000 |
| Stress | 0.015 | 0.015 | 1 | 5021.737 | 0.025 | 0.873 |
| Environment | 6.694 | 6.694 | 1 | 4988.474 | 11.299 | 0.001 |
| Age | 0.331 | 0.331 | 1 | 185.896 | 0.559 | 0.456 |
| Gender | 0.268 | 0.134 | 2 | 184.320 | 0.226 | 0.798 |
| Valence:Arousal | 0.850 | 0.850 | 1 | 4993.456 | 1.435 | 0.231 |

4: Task-focus

| *Row* | *Sum Sq* | *Mean Sq* | *NumDF* | *DenDF* | *F value* | *Pr(>F)* |
|---|---|---|---|---|---|---|
| Valence | 134.386 | 134.386 | 1 | 5053.732 | 190.008 | 0.000 |
| Arousal | 47.280 | 47.280 | 1 | 5048.576 | 66.850 | 0.000 |

| | | | | | | |
|---|---|---|---|---|---|---|
| Stress | 68.225 | 68.225 | 1 | 5051.923 | 96.464 | 0.000 |
| Environment | 4.560 | 4.560 | 1 | 5044.182 | 6.448 | 0.011 |
| Age | 4.079 | 4.079 | 1 | 187.572 | 5.768 | 0.017 |
| Gender | 0.375 | 0.188 | 2 | 184.623 | 0.265 | 0.767 |
| Valence:Arousal | 6.562 | 6.562 | 1 | 5047.635 | 9.279 | 0.002 |

| | **Future-self** | | | | | **Intrusive distraction** | | | | | **Sensory engagement** | | | | | **Task-focus** | | | | |
|---|---|---|---|---|---|---|---|---|---|---|---|---|---|---|---|---|---|---|---|---|
| *Predictors* | *Estimates* | *CI* | *Statistic* | *p* | *df* | *Estimates* | *CI* | *Statistic* | *p* | *df* | *Estimates* | *CI* | *Statistic* | *p* | *df* | *Estimates* | *CI* | *Statistic* | *p* | *df* |
| (Intercept) | 0.07 | -0.09 – 0.23 | 0.85 | 0.394 | 5052.00 | 0.19 | 0.01 – 0.36 | 2.06 | **0.040** | 5052.00 | -0.03 | -0.25 – 0.20 | -0.23 | 0.818 | 5052.00 | -0.00 | -0.18 – 0.17 | -0.04 | 0.965 | 5052.00 |
| Valence | 0.15 | 0.12 – 0.18 | 10.34 | **<0.001** | 5052.00 | -0.24 | -0.27 – -0.21 | -17.31 | **<0.001** | 5052.00 | 0.20 | 0.17 – 0.23 | 13.78 | **<0.001** | 5052.00 | 0.22 | 0.19 – 0.25 | 13.78 | **<0.001** | 5052.00 |
| Arousal | 0.30 | 0.28 – 0.33 | 22.70 | **<0.001** | 5052.00 | 0.16 | 0.13 – 0.18 | 12.12 | **<0.001** | 5052.00 | 0.17 | 0.15 – 0.20 | 12.95 | **<0.001** | 5052.00 | 0.12 | 0.09 – 0.15 | 8.18 | **<0.001** | 5052.00 |
| Stress | 0.25 | 0.22 – 0.28 | 17.02 | **<0.001** | 5052.00 | 0.22 | 0.19 – 0.25 | 15.53 | **<0.001** | 5052.00 | -0.00 | -0.03 – 0.03 | -0.16 | 0.873 | 5052.00 | 0.16 | 0.13 – 0.19 | 9.82 | **<0.001** | 5052.00 |
| Environment1 | 0.02 | 0.00 – 0.05 | 2.06 | **0.039** | 5052.00 | -0.07 | -0.09 – -0.04 | -5.65 | **<0.001** | 5052.00 | 0.04 | 0.02 – 0.07 | 3.36 | **0.001** | 5052.00 | 0.03 | 0.01 – 0.06 | 2.54 | **0.011** | 5052.00 |
| Age | 0.14 | 0.08 – 0.19 | 4.67 | **<0.001** | 5052.00 | 0.03 | -0.03 – 0.10 | 1.03 | 0.301 | 5052.00 | -0.03 | -0.11 – 0.05 | -0.75 | 0.455 | 5052.00 | 0.08 | 0.01 – 0.14 | 2.40 | **0.016** | 5052.00 |

| | | | | | | | | | | | | | | | | | | | | |
|---|---|---|---|---|---|---|---|---|---|---|---|---|---|---|---|---|---|---|---|---|
| Gender1 | -0.07 | -0.24 – 0.09 | -0.85 | 0.395 | 5052.00 | -0.18 | -0.36 – -0.00 | -1.96 | **0.050** | 5052.00 | 0.02 | -0.21 – 0.25 | 0.14 | 0.885 | 5052.00 | -0.02 | -0.20 – 0.17 | -0.16 | 0.870 | 5052.00 |
| Gender2 | -0.10 | -0.28 – 0.08 | -1.07 | 0.284 | 5052.00 | -0.02 | -0.22 – 0.18 | -0.20 | 0.840 | 5052.00 | 0.08 | -0.17 – 0.34 | 0.62 | 0.534 | 5052.00 | -0.07 | -0.27 – 0.13 | -0.68 | 0.498 | 5052.00 |
| Valence × Arousal | 0.01 | -0.01 – 0.03 | 0.89 | 0.376 | 5052.00 | -0.07 | -0.09 – -0.05 | -6.55 | **<0.001** | 5052.00 | 0.01 | -0.01 – 0.03 | 1.20 | 0.231 | 5052.00 | 0.03 | 0.01 – 0.06 | 3.05 | **0.002** | 5052.00 |
| **Random Effects** | | | | | | | | | | | | | | | | | | | | |
| $\sigma^2$ | 0.58 | | | | | 0.54 | | | | | 0.59 | | | | | 0.71 | | | | |
| $\tau_{00}$ | 0.14 participant_code | | | | | 0.18 participant_code | | | | | 0.29 participant_code | | | | | 0.17 participant_code | | | | |
| N | 190 participant_code | | | | | 190 participant_code | | | | | 190 participant_code | | | | | 190 participant_code | | | | |
| Observations | 5063 | | | | | 5063 | | | | | 5063 | | | | | 5063 | | | | |
| Marginal $R^2$ / Conditional $R^2$ | 0.243 / 0.390 | | | | | 0.233 / 0.420 | | | | | 0.093 / 0.392 | | | | | 0.099 / 0.273 | | | | |

**Supplementary Table 7.** ANOVA outputs and LMM summary tables for the main effect of TAS-20 total score on 4 thought dimensions

1: Future-self

| *Row* | *Sum Sq* | *Mean Sq* | *NumDF* | *DenDF* | *F value* | *Pr(>F)* |
|---|---|---|---|---|---|---|
| TAS_total | 4.752 | 4.752 | 1 | 184.859 | 6.554 | 0.011 |
| DASS_total | 8.312 | 8.312 | 1 | 185.236 | 11.462 | 0.001 |
| Age | 12.377 | 12.377 | 1 | 185.638 | 17.069 | 0.000 |
| Gender | 0.427 | 0.214 | 2 | 183.829 | 0.295 | 0.745 |

2: Intrusive distraction

| *Row* | *Sum Sq* | *Mean Sq* | *NumDF* | *DenDF* | *F value* | *Pr(>F)* |
|---|---|---|---|---|---|---|
| TAS_total | 2.062 | 2.062 | 1 | 183.670 | 3.005 | 0.085 |
| DASS_total | 25.126 | 25.126 | 1 | 184.025 | 36.615 | 0.000 |
| Age | 0.842 | 0.842 | 1 | 184.380 | 1.227 | 0.270 |
| Gender | 4.651 | 2.325 | 2 | 182.724 | 3.389 | 0.036 |

3: Sensory engagement

| *Row* | *Sum Sq* | *Mean Sq* | *NumDF* | *DenDF* | *F value* | *Pr(>F)* |
|---|---|---|---|---|---|---|
| TAS_total | 0.009 | 0.009 | 1 | 184.075 | 0.013 | 0.909 |
| DASS_total | 2.333 | 2.333 | 1 | 184.349 | 3.563 | 0.061 |
| Age | 0.016 | 0.016 | 1 | 184.566 | 0.025 | 0.874 |

| | | | | | | |
|---|---|---|---|---|---|---|
| Gender | 1.190 | 0.595 | 2 | 183.409 | 0.909 | 0.405 |

4: Task-focus

| *Row* | *Sum Sq* | *Mean Sq* | *NumDF* | *DenDF* | *F value* | *Pr(>F)* |
|---|---|---|---|---|---|---|
| TAS_total | 0.000 | 0.000 | 1 | 184.904 | 0.000 | 0.984 |
| DASS_total | 0.046 | 0.046 | 1 | 185.295 | 0.061 | 0.806 |
| Age | 5.311 | 5.311 | 1 | 185.736 | 6.940 | 0.009 |
| Gender | 0.017 | 0.008 | 2 | 183.809 | 0.011 | 0.989 |

| | **Future-self** | | | | | **Intrusive distraction** | | | | | **Sensory engagement** | | | | | **Task-focus** | | | | |
|---|---|---|---|---|---|---|---|---|---|---|---|---|---|---|---|---|---|---|---|---|
| *Predictors* | *Estimates* | *CI* | *Statistic* | *p* | *df* | *Estimates* | *CI* | *Statistic* | *p* | *df* | *Estimates* | *CI* | *Statistic* | *p* | *df* | *Estimates* | *CI* | *Statistic* | *p* | *df* |
| (Intercept) | 0.01 | -0.20 – 0.21 | 0.07 | 0.944 | 5055.00 | 0.11 | -0.10 – 0.32 | 1.06 | 0.289 | 5055.00 | -0.09 | -0.33 – 0.16 | -0.69 | 0.488 | 5055.00 | -0.02 | -0.22 – 0.19 | -0.16 | 0.872 | 5055.00 |
| TAS total | -0.12 | -0.21 – -0.03 | -2.56 | **0.010** | 5055.00 | -0.08 | -0.18 – 0.01 | -1.73 | 0.083 | 5055.00 | -0.01 | -0.12 – 0.10 | -0.11 | 0.909 | 5055.00 | -0.00 | -0.09 – 0.09 | -0.02 | 0.984 | 5055.00 |
| DASS total | 0.15 | 0.06 – 0.24 | 3.39 | **0.001** | 5055.00 | 0.28 | 0.19 – 0.37 | 6.05 | **<0.001** | 5055.00 | 0.10 | -0.00 – 0.21 | 1.89 | 0.059 | 5055.00 | -0.01 | -0.10 – 0.08 | -0.25 | 0.805 | 5055.00 |
| Age | 0.16 | 0.08 – 0.24 | 4.13 | **<0.001** | 5055.00 | 0.04 | -0.03 – 0.12 | 1.11 | 0.268 | 5055.00 | 0.01 | -0.08 – 0.10 | 0.16 | 0.874 | 5055.00 | 0.10 | 0.03 – 0.18 | 2.63 | **0.008** | 5055.00 |
| Gender1 | -0.01 | -0.22 – 0.20 | -0.11 | 0.911 | 5055.00 | -0.15 | -0.36 – 0.07 | -1.35 | 0.178 | 5055.00 | 0.08 | -0.17 – 0.33 | 0.65 | 0.516 | 5055.00 | 0.01 | -0.20 – 0.21 | 0.05 | 0.960 | 5055.00 |
| Gender2 | 0.07 | -0.17 – 0.30 | 0.56 | 0.577 | 5055.00 | 0.13 | -0.11 – 0.36 | 1.05 | 0.294 | 5055.00 | 0.19 | -0.09 – 0.47 | 1.34 | 0.180 | 5055.00 | -0.01 | -0.24 – 0.22 | -0.08 | 0.932 | 5055.00 |

**Random Effects**

| | | | | |
|---|---|---|---|---|
| $\sigma^2$ | 0.73 | 0.69 | 0.65 | 0.77 |
| $\tau_{00}$ | 0.23 participant_code | 0.24 participant_code | 0.34 participant_code | 0.23 participant_code |
| N | 190 participant_code | 190 participant_code | 190 participant_code | 190 participant_code |
| Observations | 5063 | 5063 | 5063 | 5063 |
| Marginal $R^2$ / Conditional $R^2$ | 0.049 / 0.279 | 0.068 / 0.312 | 0.011 / 0.352 | 0.011 / 0.237 |

**Supplementary Table 8.** ANOVA outputs and LMM summary tables for the main effect of TAS-20 subscales on 4 thought dimensions

1: Future-self

| *Row* | *Sum Sq* | *Mean Sq* | *NumDF* | *DenDF* | *F value* | *Pr(>F)* |
|---|---|---|---|---|---|---|
| TAS_DIF | 0.937 | 0.937 | 1 | 183.026 | 1.293 | 0.257 |
| TAS_DDF | 3.196 | 3.196 | 1 | 183.289 | 4.407 | 0.037 |
| TAS_EOT | 0.060 | 0.060 | 1 | 182.438 | 0.082 | 0.775 |
| DASS_total | 8.716 | 8.716 | 1 | 183.560 | 12.020 | 0.001 |
| Age | 13.031 | 13.031 | 1 | 183.965 | 17.970 | 0.000 |
| Gender | 0.465 | 0.232 | 2 | 181.845 | 0.320 | 0.726 |

2: Intrusive distraction

| *Row* | *Sum Sq* | *Mean Sq* | *NumDF* | *DenDF* | *F value* | *Pr(>F)* |
|---|---|---|---|---|---|---|
| TAS_DIF | 0.082 | 0.082 | 1 | 181.486 | 0.120 | 0.729 |
| TAS_DDF | 3.392 | 3.392 | 1 | 181.729 | 4.942 | 0.027 |
| TAS_EOT | 0.440 | 0.440 | 1 | 180.945 | 0.642 | 0.424 |
| DASS_total | 24.654 | 24.654 | 1 | 181.985 | 35.925 | 0.000 |
| Age | 1.043 | 1.043 | 1 | 182.349 | 1.519 | 0.219 |
| Gender | 4.838 | 2.419 | 2 | 180.393 | 3.525 | 0.031 |

3: Sensory engagement

| *Row* | *Sum Sq* | *Mean Sq* | *NumDF* | *DenDF* | *F value* | *Pr(>F)* |
|---|---|---|---|---|---|---|
| TAS_DIF | 0.449 | 0.449 | 1 | 181.998 | 0.686 | 0.409 |
| TAS_DDF | 0.850 | 0.850 | 1 | 182.168 | 1.298 | 0.256 |
| TAS_EOT | 0.043 | 0.043 | 1 | 181.629 | 0.065 | 0.799 |
| DASS_total | 1.799 | 1.799 | 1 | 182.361 | 2.748 | 0.099 |
| Age | 0.007 | 0.007 | 1 | 182.586 | 0.010 | 0.919 |
| Gender | 1.651 | 0.825 | 2 | 181.233 | 1.261 | 0.286 |

4: Task-focus

| *Row* | *Sum Sq* | *Mean Sq* | *NumDF* | *DenDF* | *F value* | *Pr(>F)* |
|---|---|---|---|---|---|---|
| TAS_DIF | 1.215 | 1.215 | 1 | 182.983 | 1.588 | 0.209 |
| TAS_DDF | 1.686 | 1.686 | 1 | 183.262 | 2.203 | 0.139 |
| TAS_EOT | 0.060 | 0.060 | 1 | 182.356 | 0.079 | 0.779 |
| DASS_total | 0.172 | 0.172 | 1 | 183.545 | 0.225 | 0.636 |
| Age | 4.819 | 4.819 | 1 | 183.986 | 6.298 | 0.013 |
| Gender | 0.077 | 0.038 | 2 | 181.730 | 0.050 | 0.951 |

| | **Future-self** | | | | | **Intrusive distraction** | | | | | **Sensory engagement** | | | | | **Task-focus** | | | | |
|---|---|---|---|---|---|---|---|---|---|---|---|---|---|---|---|---|---|---|---|---|
| *Predictors* | *Estimates* | *CI* | *Statistic* | *p* | *df* | *Estimates* | *CI* | *Statistic* | *p* | *df* | *Estimates* | *CI* | *Statistic* | *p* | *df* | *Estimates* | *CI* | *Statistic* | *p* | *df* |
| (Intercept) | -0.00 | -0.21 – 0.20 | -0.01 | 0.992 | 5053.00 | 0.10 | -0.11 – 0.31 | 0.95 | 0.341 | 5053.00 | -0.10 | -<br>0.34 – 0.15 | -0.76 | 0.445 | 5053.00 | -0.03 | -<br>0.23 – 0.18 | -0.26 | 0.795 | 5053.00 |

| | | | | | | | | | | | | | | | | | | | | |
|---|---|---|---|---|---|---|---|---|---|---|---|---|---|---|---|---|---|---|---|---|
| TAS DIF | -0.06 | -0.16 – 0.04 | -1.14 | 0.256 | 5053.00 | -0.02 | -0.12 – 0.09 | -0.35 | 0.729 | 5053.00 | 0.05 | -0.07 – 0.18 | 0.83 | 0.407 | 5053.00 | 0.07 | -0.04 – 0.17 | 1.26 | 0.208 | 5053.00 |
| TAS DDF | -0.10 | -0.19 – -0.01 | -2.10 | 0.036 | 5053.00 | -0.10 | -0.20 – -0.01 | -2.22 | 0.026 | 5053.00 | -0.06 | -0.17 – 0.05 | -1.14 | 0.255 | 5053.00 | -0.07 | -0.16 – 0.02 | -1.48 | 0.138 | 5053.00 |
| TAS EOT | 0.01 | -0.07 – 0.09 | 0.29 | 0.774 | 5053.00 | 0.03 | -0.05 – 0.11 | 0.80 | 0.423 | 5053.00 | 0.01 | -0.08 – 0.10 | 0.26 | 0.798 | 5053.00 | 0.01 | -0.07 – 0.09 | 0.28 | 0.779 | 5053.00 |
| DASS total | 0.16 | 0.07 – 0.26 | 3.47 | **0.001** | 5053.00 | 0.29 | 0.19 – 0.38 | 5.99 | **<0.001** | 5053.00 | 0.09 | -0.02 – 0.21 | 1.66 | 0.097 | 5053.00 | -0.02 | -0.11 – 0.07 | -0.47 | 0.635 | 5053.00 |
| Age | 0.17 | 0.09 – 0.24 | 4.24 | **<0.001** | 5053.00 | 0.05 | -0.03 – 0.13 | 1.23 | 0.218 | 5053.00 | 0.00 | -0.09 – 0.10 | 0.10 | 0.919 | 5053.00 | 0.10 | 0.02 – 0.17 | 2.51 | **0.01**2 | 5053.00 |
| Gender1 | -0.00 | -0.21 – 0.21 | -0.02 | 0.984 | 5053.00 | -0.13 | -0.35 – 0.08 | -1.24 | 0.215 | 5053.00 | 0.09 | -0.16 – 0.34 | 0.69 | 0.487 | 5053.00 | 0.01 | -0.20 – 0.22 | 0.11 | 0.913 | 5053.00 |
| Gender2 | 0.08 | -0.16 – 0.32 | 0.65 | 0.515 | 5053.00 | 0.15 | -0.09 – 0.40 | 1.25 | 0.212 | 5053.00 | 0.23 | -0.06 – 0.52 | 1.57 | 0.116 | 5053.00 | 0.04 | -0.20 – 0.28 | 0.31 | 0.759 | 5053.00 |
| **Random Effects** | | | | | | | | | | | | | | | | | | | | |
| $\sigma^2$ | 0.73 | | | | | 0.69 | | | | | 0.65 | | | | | 0.77 | | | | |
| $\tau_{00}$ | 0.23 participant_code | | | | | 0.24 participant_code | | | | | 0.34 participant_code | | | | | 0.23 participant_code | | | | |
| N | 190 participant_code | | | | | 190 participant_code | | | | | 190 participant_code | | | | | 190 participant_code | | | | |
| Observations | 5063 | | | | | 5063 | | | | | 5063 | | | | | 5063 | | | | |
| Marginal $R^2$ / Conditional $R^2$ | 0.053 / 0.281 | | | | | 0.074 / 0.314 | | | | | 0.014 / 0.354 | | | | | 0.014 / 0.239 | | | | |

**Supplementary Table 9.** ANOVA outputs and LMM summary tables for the interaction effects of TAS-20 total score and affective states on 4 thought dimensions

1: Future-self

| *Row* | *Sum Sq* | *Mean Sq* | *NumDF* | *DenDF* | *F value* | *Pr(>F)* |
|---|---|---|---|---|---|---|
| TAS_total | 2.590 | 2.590 | 1 | 183.674 | 4.427 | 0.037 |
| Valence | 63.283 | 63.283 | 1 | 5047.395 | 108.172 | 0.000 |
| Arousal | 300.831 | 300.831 | 1 | 5042.601 | 514.222 | 0.000 |
| Stress | 165.109 | 165.109 | 1 | 5047.934 | 282.227 | 0.000 |
| Environment | 2.280 | 2.280 | 1 | 5040.317 | 3.898 | 0.048 |
| DASS_total | 2.137 | 2.137 | 1 | 187.631 | 3.653 | 0.057 |
| Age | 8.940 | 8.940 | 1 | 185.938 | 15.282 | 0.000 |
| Gender | 0.506 | 0.253 | 2 | 181.919 | 0.433 | 0.649 |
| Valence:Arousal | 0.292 | 0.292 | 1 | 5040.130 | 0.500 | 0.480 |
| TAS_total:Valence | 0.296 | 0.296 | 1 | 5047.927 | 0.506 | 0.477 |
| TAS_total:Arousal | 1.496 | 1.496 | 1 | 4928.524 | 2.557 | 0.110 |
| TAS_total:Stress | 0.081 | 0.081 | 1 | 5045.599 | 0.139 | 0.709 |
| TAS_total:Valence:Arousal | 0.380 | 0.380 | 1 | 5045.784 | 0.650 | 0.420 |

2: Intrusive distraction

| *Row* | *Sum Sq* | *Mean Sq* | *NumDF* | *DenDF* | *F value* | *Pr(>F)* |
|---|---|---|---|---|---|---|
| TAS_total | 0.826 | 0.826 | 1 | 181.465 | 1.521 | 0.219 |

| | | | | | | |
|---|---|---|---|---|---|---|
| Valence | 160.891 | 160.891 | 1 | 5044.308 | 296.002 | 0.000 |
| Arousal | 79.499 | 79.499 | 1 | 5047.654 | 146.260 | 0.000 |
| Stress | 126.716 | 126.716 | 1 | 5042.303 | 233.128 | 0.000 |
| Environment | 18.012 | 18.012 | 1 | 5024.731 | 33.138 | 0.000 |
| DASS_total | 8.582 | 8.582 | 1 | 184.698 | 15.789 | 0.000 |
| Age | 0.659 | 0.659 | 1 | 183.329 | 1.213 | 0.272 |
| Gender | 2.967 | 1.484 | 2 | 180.031 | 2.730 | 0.068 |
| Valence:Arousal | 22.778 | 22.778 | 1 | 5024.630 | 41.906 | 0.000 |
| TAS_total:Valence | 0.267 | 0.267 | 1 | 5042.430 | 0.491 | 0.484 |
| TAS_total:Arousal | 0.001 | 0.001 | 1 | 5001.985 | 0.002 | 0.966 |
| TAS_total:Stress | 0.112 | 0.112 | 1 | 5046.303 | 0.207 | 0.649 |
| TAS_total:Valence:Arousal | 0.001 | 0.001 | 1 | 5046.370 | 0.002 | 0.966 |

## 3: Sensory engagement

| *Row* | *Sum Sq* | *Mean Sq* | *NumDF* | *DenDF* | *F value* | *Pr(>F)* |
|---|---|---|---|---|---|---|
| TAS_total | 0.016 | 0.016 | 1 | 183.278 | 0.027 | 0.871 |
| Valence | 111.916 | 111.916 | 1 | 5013.330 | 189.357 | 0.000 |
| Arousal | 103.051 | 103.051 | 1 | 5023.518 | 174.357 | 0.000 |
| Stress | 0.076 | 0.076 | 1 | 5009.171 | 0.128 | 0.721 |
| Environment | 5.870 | 5.870 | 1 | 4987.403 | 9.932 | 0.002 |
| DASS_total | 3.323 | 3.323 | 1 | 185.491 | 5.622 | 0.019 |

| | | | | | | |
|---|---|---|---|---|---|---|
| Age | 0.047 | 0.047 | 1 | 184.570 | 0.080 | 0.777 |
| Gender | 0.689 | 0.345 | 2 | 182.297 | 0.583 | 0.559 |
| Valence:Arousal | 0.744 | 0.744 | 1 | 4987.551 | 1.259 | 0.262 |
| TAS_total:Valence | 3.989 | 3.989 | 1 | 5009.590 | 6.749 | 0.009 |
| TAS_total:Arousal | 1.203 | 1.203 | 1 | 5047.972 | 2.035 | 0.154 |
| TAS_total:Stress | 0.058 | 0.058 | 1 | 5017.781 | 0.098 | 0.754 |
| TAS_total:Valence:Arousal | 1.618 | 1.618 | 1 | 5018.792 | 2.737 | 0.098 |

4: Task-focus

| *Row* | *Sum Sq* | *Mean Sq* | *NumDF* | *DenDF* | *F value* | *Pr(>F)* |
|---|---|---|---|---|---|---|
| TAS_total | 0.151 | 0.151 | 1 | 184.567 | 0.213 | 0.645 |
| Valence | 133.285 | 133.285 | 1 | 5047.904 | 188.361 | 0.000 |
| Arousal | 45.815 | 45.815 | 1 | 5044.703 | 64.746 | 0.000 |
| Stress | 68.437 | 68.437 | 1 | 5047.960 | 96.717 | 0.000 |
| Environment | 4.815 | 4.815 | 1 | 5038.290 | 6.804 | 0.009 |
| DASS_total | 0.406 | 0.406 | 1 | 188.424 | 0.574 | 0.450 |
| Age | 3.892 | 3.892 | 1 | 186.777 | 5.501 | 0.020 |
| Gender | 0.479 | 0.240 | 2 | 182.857 | 0.339 | 0.713 |
| Valence:Arousal | 6.929 | 6.929 | 1 | 5038.110 | 9.792 | 0.002 |
| TAS_total:Valence | 0.031 | 0.031 | 1 | 5047.966 | 0.044 | 0.834 |
| TAS_total:Arousal | 0.716 | 0.716 | 1 | 4944.110 | 1.012 | 0.314 |

| | | | | | | |
|---|---|---|---|---|---|---|
| TAS_total:Stress | 0.000 | 0.000 | 1 | 5046.893 | 0.000 | 0.984 |
| TAS_total:Valence:Arousal | 0.362 | 0.362 | 1 | 5046.982 | 0.511 | 0.475 |

| | **Future-self** | | | | | **Intrusive distraction** | | | | | **Sensory engagement** | | | | | **Task-focus** | | | | |
|---|---|---|---|---|---|---|---|---|---|---|---|---|---|---|---|---|---|---|---|---|
| *Predictors* | *Estimates* | *CI* | *Statistic* | *p* | *df* | *Estimates* | *CI* | *Statistic* | *p* | *df* | *Estimates* | *CI* | *Statistic* | *p* | *df* | *Estimates* | *CI* | *Statistic* | *p* | *df* |
| (Intercept) | 0.06 | -0.10 – 0.22 | 0.72 | 0.474 | 5046.00 | 0.15 | -0.02 – 0.32 | 1.74 | 0.082 | 5046.00 | -0.05 | -0.27 – 0.17 | -0.43 | 0.664 | 5046.00 | 0.00 | -0.18 – 0.18 | 0.00 | 0.999 | 5046.00 |
| Valence | 0.15 | 0.12 – 0.18 | 10.40 | **<0.001** | 5046.00 | -0.24 | -0.26 – -0.21 | -17.20 | **<0.001** | 5046.00 | 0.20 | 0.17 – 0.23 | 13.76 | **<0.001** | 5046.00 | 0.22 | 0.18 – 0.25 | 13.72 | **<0.001** | 5046.00 |
| Arousal | 0.30 | 0.28 – 0.33 | 22.68 | **<0.001** | 5046.00 | 0.16 | 0.13 – 0.18 | 12.09 | **<0.001** | 5046.00 | 0.18 | 0.15 – 0.21 | 13.20 | **<0.001** | 5046.00 | 0.12 | 0.09 – 0.15 | 8.05 | **<0.001** | 5046.00 |
| TAS total | -0.08 | -0.15 – -0.01 | -2.10 | **0.035** | 5046.00 | -0.05 | -0.13 – 0.03 | -1.23 | 0.218 | 5046.00 | 0.01 | -0.09 – 0.11 | 0.16 | 0.871 | 5046.00 | 0.02 | -0.06 – 0.10 | 0.46 | 0.645 | 5046.00 |
| Stress | 0.25 | 0.22 – 0.27 | 16.80 | **<0.001** | 5046.00 | 0.22 | 0.19 – 0.24 | 15.27 | **<0.001** | 5046.00 | -0.01 | -0.03 – 0.02 | -0.36 | 0.721 | 5046.00 | 0.16 | 0.13 – 0.19 | 9.83 | **<0.001** | 5046.00 |
| Environment1 | 0.02 | 0.00 – 0.05 | 1.97 | 0.048 | 5046.00 | -0.07 | -0.09 – -0.04 | -5.76 | **<0.001** | 5046.00 | 0.04 | 0.01 – 0.06 | 3.15 | **0.002** | 5046.00 | 0.03 | 0.01 – 0.06 | 2.61 | **0.009** | 5046.00 |
| DASS total | 0.07 | -0.00 – 0.14 | 1.91 | 0.056 | 5046.00 | 0.15 | 0.08 – 0.23 | 3.97 | **<0.001** | 5046.00 | 0.12 | 0.02 – 0.21 | 2.37 | **0.018** | 5046.00 | -0.03 | -0.11 – 0.05 | -0.76 | 0.449 | 5046.00 |
| Age | 0.12 | 0.06 – 0.18 | 3.91 | **<0.001** | 5046.00 | 0.04 | -0.03 – 0.10 | 1.10 | 0.271 | 5046.00 | -0.01 | -0.09 – 0.07 | -0.28 | 0.777 | 5046.00 | 0.08 | 0.01 – 0.15 | 2.35 | **0.019** | 5046.00 |
| Gender1 | -0.06 | -0.23 – 0.10 | -0.75 | 0.456 | 5046.00 | -0.15 | -0.33 – 0.03 | -1.67 | 0.095 | 5046.00 | 0.04 | -0.19 – 0.27 | 0.34 | 0.732 | 5046.00 | -0.02 | -0.20 – 0.16 | -0.20 | 0.839 | 5046.00 |
| Gender2 | -0.08 | -0.26 – 0.10 | -0.88 | 0.377 | 5046.00 | 0.04 | -0.16 – 0.23 | 0.38 | 0.704 | 5046.00 | 0.13 | -0.12 – 0.38 | 1.03 | 0.301 | 5046.00 | -0.08 | -0.28 – 0.12 | -0.77 | 0.441 | 5046.00 |
| Valence × Arousal | 0.01 | -0.01 – 0.03 | 0.71 | 0.480 | 5046.00 | -0.06 | -0.08 – -0.05 | -6.47 | **<0.001** | 5046.00 | 0.01 | -0.01 – 0.03 | 1.12 | 0.262 | 5046.00 | 0.04 | 0.01 – 0.06 | 3.13 | **0.002** | 5046.00 |

| | | | | | | | | | | | | | | | | | | | | |
|---|---|---|---|---|---|---|---|---|---|---|---|---|---|---|---|---|---|---|---|---|
| Valence × TAS total | -0.01 | -0.04 – 0.02 | -0.71 | 0.477 | 5046.00 | 0.01 | -0.02 – 0.03 | 0.70 | 0.484 | 5046.00 | 0.04 | 0.01 – 0.06 | 2.60 | **0.009** | 5046.00 | -0.00 | -0.03 – 0.03 | -0.21 | 0.834 | 5046.00 |
| Arousal × TAS total | -0.02 | -0.05 – 0.00 | -1.60 | 0.110 | 5046.00 | -0.00 | -0.03 – 0.02 | -0.04 | 0.966 | 5046.00 | -0.02 | -0.04 – 0.01 | -1.43 | 0.154 | 5046.00 | 0.01 | -0.01 – 0.04 | 1.01 | 0.314 | 5046.00 |
| TAS total × Stress | 0.01 | -0.02 – 0.03 | 0.37 | 0.709 | 5046.00 | -0.01 | -0.03 – 0.02 | -0.45 | 0.649 | 5046.00 | 0.00 | -0.02 – 0.03 | 0.31 | 0.754 | 5046.00 | -0.00 | -0.03 – 0.03 | -0.02 | 0.984 | 5046.00 |
| (Valence × Arousal) × TAS total | 0.01 | -0.01 – 0.03 | 0.81 | 0.420 | 5046.00 | 0.00 | -0.02 – 0.02 | 0.04 | 0.966 | 5046.00 | 0.02 | -0.00 – 0.03 | 1.65 | 0.098 | 5046.00 | -0.01 | -0.03 – 0.01 | -0.72 | 0.475 | 5046.00 |
| **Random Effects** | | | | | | | | | | | | | | | | | | | | |
| $\sigma^2$ | 0.59 | | | | | 0.54 | | | | | 0.59 | | | | | 0.71 | | | | |
| $\tau_{00}$ | 0.14 participant_code | | | | | 0.16 participant_code | | | | | 0.28 participant_code | | | | | 0.17 participant_code | | | | |
| N | 190 participant_code | | | | | 190 participant_code | | | | | 190 participant_code | | | | | 190 participant_code | | | | |
| Observations | 5063 | | | | | 5063 | | | | | 5063 | | | | | 5063 | | | | |
| Marginal $R^2$ / Conditional $R^2$ | 0.254 / 0.394 | | | | | 0.269 / 0.436 | | | | | 0.104 / 0.391 | | | | | 0.098 / 0.274 | | | | |

## Post-hoc Contrasts for PC3: Sensory Engagement (TAS-total x Valence)

| *Contrast* | *estimate* | *SE* | *df* | *t* | *p.value* | *conf.low* | *conf.high* |
|---|---|---|---|---|---|---|---|
| LowTAS: Valence High – Low | -0.5307 | 0.1655 | 5017.7445 | -3.2068 | 0.0014 | -0.8552 | -0.2063 |
| HighTAS: Valence High – Low | -1.3155 | 0.1645 | 5001.3826 | -7.9977 | 0.0000 | -1.6380 | -0.9930 |
| Contrast of contrasts: (Valence High – Low) HighTAS vs LowTAS | 0.7848 | 0.3021 | 5009.5904 | 2.5979 | 0.0094 | 0.1926 | 1.3770 |

**Supplementary Table 10.** ANOVA outputs and LMM summary tables (with post-hoc contrasts) for the interaction effects of TAS-20 subscales and affective states on 4 thought dimensions

1: Future-self

| *Row* | *Sum Sq* | *Mean Sq* | *NumDF* | *DenDF* | *F value* | *Pr(>F)* |
|---|---|---|---|---|---|---|
| TAS_DIF | 0.805 | 0.805 | 1 | 180.432 | 1.378 | 0.242 |
| TAS_DDF | 0.959 | 0.959 | 1 | 181.272 | 1.642 | 0.202 |
| TAS_EOT | 0.001 | 0.001 | 1 | 184.031 | 0.002 | 0.963 |
| Valence | 59.471 | 59.471 | 1 | 5037.193 | 101.809 | 0.000 |
| Arousal | 301.789 | 301.789 | 1 | 5029.532 | 516.638 | 0.000 |
| Stress | 161.576 | 161.576 | 1 | 5037.928 | 276.606 | 0.000 |
| Environment | 2.607 | 2.607 | 1 | 5029.400 | 4.463 | 0.035 |
| DASS_total | 2.320 | 2.320 | 1 | 186.596 | 3.972 | 0.048 |
| Age | 8.986 | 8.986 | 1 | 183.924 | 15.383 | 0.000 |
| Gender | 0.457 | 0.229 | 2 | 179.176 | 0.391 | 0.677 |
| Valence:Arousal | 0.019 | 0.019 | 1 | 5032.008 | 0.032 | 0.858 |
| TAS_DIF:Valence | 0.187 | 0.187 | 1 | 5021.852 | 0.321 | 0.571 |
| TAS_DIF:Arousal | 1.315 | 1.315 | 1 | 5035.509 | 2.252 | 0.134 |
| TAS_DIF:Stress | 0.297 | 0.297 | 1 | 5025.249 | 0.508 | 0.476 |
| TAS_DDF:Valence | 1.885 | 1.885 | 1 | 5034.853 | 3.228 | 0.072 |
| TAS_DDF:Arousal | 0.055 | 0.055 | 1 | 5013.805 | 0.095 | 0.758 |
| TAS_DDF:Stress | 0.101 | 0.101 | 1 | 5036.667 | 0.172 | 0.678 |

| | | | | | | |
|---|---|---|---|---|---|---|
| TAS_EOT:Valence | 0.380 | 0.380 | 1 | 5035.454 | 0.651 | 0.420 |
| TAS_EOT:Arousal | 0.034 | 0.034 | 1 | 5037.944 | 0.059 | 0.808 |
| TAS_EOT:Stress | 0.002 | 0.002 | 1 | 5036.873 | 0.003 | 0.958 |
| TAS_DIF:Valence:Arousal | 4.178 | 4.178 | 1 | 5019.014 | 7.153 | 0.008 |
| TAS_DDF:Valence:Arousal | 0.530 | 0.530 | 1 | 5037.466 | 0.907 | 0.341 |
| TAS_EOT:Valence:Arousal | 1.487 | 1.487 | 1 | 5030.230 | 2.546 | 0.111 |

2: Intrusive distraction

| *Row* | *Sum Sq* | *Mean Sq* | *NumDF* | *DenDF* | *F value* | *Pr(>F)* |
|---|---|---|---|---|---|---|
| TAS_DIF | 0.007 | 0.007 | 1 | 179.471 | 0.014 | 0.907 |
| TAS_DDF | 1.233 | 1.233 | 1 | 180.168 | 2.274 | 0.133 |
| TAS_EOT | 0.037 | 0.037 | 1 | 182.425 | 0.068 | 0.795 |
| Valence | 158.895 | 158.895 | 1 | 5035.015 | 293.088 | 0.000 |
| Arousal | 76.840 | 76.840 | 1 | 5038.000 | 141.736 | 0.000 |
| Stress | 122.749 | 122.749 | 1 | 5030.514 | 226.416 | 0.000 |
| Environment | 17.389 | 17.389 | 1 | 5014.083 | 32.074 | 0.000 |
| DASS_total | 8.046 | 8.046 | 1 | 184.551 | 14.842 | 0.000 |
| Age | 0.690 | 0.690 | 1 | 182.375 | 1.272 | 0.261 |
| Gender | 3.083 | 1.542 | 2 | 178.436 | 2.844 | 0.061 |
| Valence:Arousal | 20.010 | 20.010 | 1 | 5018.177 | 36.910 | 0.000 |
| TAS_DIF:Valence | 0.905 | 0.905 | 1 | 5037.187 | 1.670 | 0.196 |

| | | | | | | |
|---|---|---|---|---|---|---|
| TAS_DIF:Arousal | 0.349 | 0.349 | 1 | 5036.775 | 0.644 | 0.422 |
| TAS_DIF:Stress | 0.011 | 0.011 | 1 | 5037.789 | 0.020 | 0.887 |
| TAS_DDF:Valence | 0.174 | 0.174 | 1 | 5037.054 | 0.320 | 0.572 |
| TAS_DDF:Arousal | 0.084 | 0.084 | 1 | 5035.029 | 0.155 | 0.694 |
| TAS_DDF:Stress | 0.628 | 0.628 | 1 | 5035.716 | 1.158 | 0.282 |
| TAS_EOT:Valence | 3.719 | 3.719 | 1 | 5023.732 | 6.860 | 0.009 |
| TAS_EOT:Arousal | 0.291 | 0.291 | 1 | 5030.869 | 0.538 | 0.463 |
| TAS_EOT:Stress | 0.517 | 0.517 | 1 | 5035.373 | 0.954 | 0.329 |
| TAS_DIF:Valence:Arousal | 0.129 | 0.129 | 1 | 5001.373 | 0.239 | 0.625 |
| TAS_DDF:Valence:Arousal | 1.984 | 1.984 | 1 | 5028.814 | 3.660 | 0.056 |
| TAS_EOT:Valence:Arousal | 3.625 | 3.625 | 1 | 5037.960 | 6.687 | 0.010 |

3: Sensory engagement

| *Row* | *Sum Sq* | *Mean Sq* | *NumDF* | *DenDF* | *F value* | *Pr(>F)* |
|---|---|---|---|---|---|---|
| TAS_DIF | 0.574 | 0.574 | 1 | 180.437 | 0.970 | 0.326 |
| TAS_DDF | 0.682 | 0.682 | 1 | 180.915 | 1.153 | 0.284 |
| TAS_EOT | 0.147 | 0.147 | 1 | 182.427 | 0.249 | 0.618 |
| Valence | 108.908 | 108.908 | 1 | 5005.041 | 184.234 | 0.000 |
| Arousal | 100.306 | 100.306 | 1 | 5017.308 | 169.681 | 0.000 |
| Stress | 0.110 | 0.110 | 1 | 4996.333 | 0.185 | 0.667 |
| Environment | 6.292 | 6.292 | 1 | 4976.616 | 10.644 | 0.001 |

| | | | | | | |
|---|---|---|---|---|---|---|
| DASS_total | 2.604 | 2.604 | 1 | 183.879 | 4.404 | 0.037 |
| Age | 0.070 | 0.070 | 1 | 182.433 | 0.118 | 0.731 |
| Gender | 1.020 | 0.510 | 2 | 179.738 | 0.863 | 0.424 |
| Valence:Arousal | 0.497 | 0.497 | 1 | 4981.663 | 0.840 | 0.359 |
| TAS_DIF:Valence | 0.894 | 0.894 | 1 | 5022.375 | 1.512 | 0.219 |
| TAS_DIF:Arousal | 0.069 | 0.069 | 1 | 5010.235 | 0.117 | 0.732 |
| TAS_DIF:Stress | 0.129 | 0.129 | 1 | 5019.352 | 0.218 | 0.640 |
| TAS_DDF:Valence | 0.013 | 0.013 | 1 | 5010.840 | 0.023 | 0.880 |
| TAS_DDF:Arousal | 0.365 | 0.365 | 1 | 5026.923 | 0.617 | 0.432 |
| TAS_DDF:Stress | 0.002 | 0.002 | 1 | 5006.714 | 0.003 | 0.959 |
| TAS_EOT:Valence | 3.621 | 3.621 | 1 | 4987.755 | 6.126 | 0.013 |
| TAS_EOT:Arousal | 0.414 | 0.414 | 1 | 4997.390 | 0.700 | 0.403 |
| TAS_EOT:Stress | 0.858 | 0.858 | 1 | 5005.501 | 1.451 | 0.228 |
| TAS_DIF:Valence:Arousal | 0.908 | 0.908 | 1 | 4964.175 | 1.536 | 0.215 |
| TAS_DDF:Valence:Arousal | 0.296 | 0.296 | 1 | 4994.939 | 0.500 | 0.479 |
| TAS_EOT:Valence:Arousal | 0.326 | 0.326 | 1 | 5015.271 | 0.552 | 0.458 |

4: Task-focus

| *Row* | *Sum Sq* | *Mean Sq* | *NumDF* | *DenDF* | *F value* | *Pr(>F)* |
|---|---|---|---|---|---|---|
| TAS_DIF | 1.651 | 1.651 | 1 | 181.715 | 2.339 | 0.128 |
| TAS_DDF | 1.030 | 1.030 | 1 | 182.544 | 1.459 | 0.229 |

| | | | | | | |
|---|---|---|---|---|---|---|
| TAS_EOT | 0.037 | 0.037 | 1 | 185.261 | 0.052 | 0.819 |
| Valence | 132.482 | 132.482 | 1 | 5037.669 | 187.641 | 0.000 |
| Arousal | 46.852 | 46.852 | 1 | 5031.523 | 66.359 | 0.000 |
| Stress | 69.687 | 69.687 | 1 | 5037.677 | 98.702 | 0.000 |
| Environment | 4.450 | 4.450 | 1 | 5028.016 | 6.303 | 0.012 |
| DASS_total | 0.579 | 0.579 | 1 | 187.792 | 0.820 | 0.366 |
| Age | 3.403 | 3.403 | 1 | 185.161 | 4.819 | 0.029 |
| Gender | 0.074 | 0.037 | 2 | 180.476 | 0.053 | 0.949 |
| Valence:Arousal | 6.533 | 6.533 | 1 | 5030.809 | 9.253 | 0.002 |
| TAS_DIF:Valence | 1.713 | 1.713 | 1 | 5024.855 | 2.426 | 0.119 |
| TAS_DIF:Arousal | 0.172 | 0.172 | 1 | 5036.445 | 0.243 | 0.622 |
| TAS_DIF:Stress | 0.486 | 0.486 | 1 | 5027.895 | 0.689 | 0.407 |
| TAS_DDF:Valence | 0.093 | 0.093 | 1 | 5035.944 | 0.132 | 0.716 |
| TAS_DDF:Arousal | 0.429 | 0.429 | 1 | 5017.616 | 0.608 | 0.436 |
| TAS_DDF:Stress | 0.459 | 0.459 | 1 | 5037.318 | 0.651 | 0.420 |
| TAS_EOT:Valence | 4.472 | 4.472 | 1 | 5034.560 | 6.334 | 0.012 |
| TAS_EOT:Arousal | 5.909 | 5.909 | 1 | 5037.718 | 8.370 | 0.004 |
| TAS_EOT:Stress | 7.114 | 7.114 | 1 | 5037.466 | 10.076 | 0.002 |
| TAS_DIF:Valence:Arousal | 0.002 | 0.002 | 1 | 5017.329 | 0.003 | 0.954 |
| TAS_DDF:Valence:Arousal | 0.238 | 0.238 | 1 | 5036.986 | 0.337 | 0.561 |
| TAS_EOT:Valence:Arousal | 0.000 | 0.000 | 1 | 5032.176 | 0.001 | 0.980 |

| | Future-self | | | | | Intrusive distraction | | | | | Sensory engagement | | | | | Task-focus | | | | |
|---|---|---|---|---|---|---|---|---|---|---|---|---|---|---|---|---|---|---|---|---|
| *Predictors* | *Estimates* | *CI* | *Statistic* | *p* | *df* | *Estimates* | *CI* | *Statistic* | *p* | *df* | *Estimates* | *CI* | *Statistic* | *p* | *df* | *Estimates* | *CI* | *Statistic* | *p* | *df* |
| (Intercept) | 0.06 | -0.10 – 0.22 | 0.72 | 0.473 | 5036.00 | 0.14 | -0.03 – 0.32 | 1.62 | 0.105 | 5036.00 | -0.06 | -0.28 – 0.17 | -0.49 | 0.625 | 5036.00 | -0.00 | -0.18 – 0.18 | -0.03 | 0.977 | 5036.00 |
| TAS DIF | -0.05 | -0.13 – 0.03 | -1.17 | 0.240 | 5036.00 | -0.01 | -0.09 – 0.08 | -0.12 | 0.907 | 5036.00 | 0.06 | -0.06 – 0.17 | 0.99 | 0.325 | 5036.00 | 0.07 | -0.02 – 0.16 | 1.53 | 0.126 | 5036.00 |
| TAS DDF | -0.05 | -0.12 – 0.02 | -1.28 | 0.200 | 5036.00 | -0.06 | -0.14 – 0.02 | -1.51 | 0.132 | 5036.00 | -0.05 | -0.15 – 0.04 | -1.07 | 0.283 | 5036.00 | -0.05 | -0.13 – 0.03 | -1.21 | 0.227 | 5036.00 |
| TAS EOT | -0.00 | -0.06 – 0.06 | -0.05 | 0.963 | 5036.00 | 0.01 | -0.06 – 0.07 | 0.26 | 0.795 | 5036.00 | 0.02 | -0.06 – 0.10 | 0.50 | 0.618 | 5036.00 | 0.01 | -0.06 – 0.08 | 0.23 | 0.819 | 5036.00 |
| Valence | 0.14 | 0.12 – 0.17 | 10.09 | **<0.001** | 5036.00 | -0.24 | -0.26 – -0.21 | -17.12 | **<0.001** | 5036.00 | 0.20 | 0.17 – 0.23 | 13.57 | **<0.001** | 5036.00 | 0.22 | 0.18 – 0.25 | 13.70 | **<0.001** | 5036.00 |
| Arousal | 0.31 | 0.28 – 0.33 | 22.73 | **<0.001** | 5036.00 | 0.16 | 0.13 – 0.18 | 11.91 | **<0.001** | 5036.00 | 0.18 | 0.15 – 0.21 | 13.03 | **<0.001** | 5036.00 | 0.12 | 0.09 – 0.15 | 8.15 | **<0.001** | 5036.00 |
| Stress | 0.24 | 0.21 – 0.27 | 16.63 | **<0.001** | 5036.00 | 0.21 | 0.19 – 0.24 | 15.05 | **<0.001** | 5036.00 | -0.01 | -0.04 – 0.02 | -0.43 | 0.667 | 5036.00 | 0.16 | 0.13 – 0.19 | 9.93 | **<0.001** | 5036.00 |
| Environment1 | 0.03 | 0.00 – 0.05 | 2.11 | 0.035 | 5036.00 | -0.07 | -0.09 – -0.04 | -5.66 | **<0.001** | 5036.00 | 0.04 | 0.02 – 0.06 | 3.26 | **0.001** | 5036.00 | 0.03 | 0.01 – 0.06 | 2.51 | **0.012** | 5036.00 |
| DASS total | 0.08 | 0.00 – 0.15 | 1.99 | 0.046 | 5036.00 | 0.15 | 0.08 – 0.23 | 3.85 | **<0.001** | 5036.00 | 0.11 | 0.01 – 0.21 | 2.10 | 0.036 | 5036.00 | -0.04 | -0.12 – 0.04 | -0.91 | 0.365 | 5036.00 |
| Age | 0.12 | 0.06 – 0.18 | 3.92 | **<0.001** | 5036.00 | 0.04 | -0.03 – 0.10 | 1.13 | 0.259 | 5036.00 | -0.01 | -0.10 – 0.07 | -0.34 | 0.731 | 5036.00 | 0.08 | 0.01 – 0.14 | 2.20 | **0.028** | 5036.00 |
| Gender1 | -0.06 | -0.23 – 0.11 | -0.71 | 0.478 | 5036.00 | -0.14 | -0.32 – 0.03 | -1.60 | 0.110 | 5036.00 | 0.04 | -0.18 – 0.27 | 0.38 | 0.705 | 5036.00 | -0.02 | -0.20 – 0.16 | -0.21 | 0.837 | 5036.00 |
| Gender2 | -0.08 | -0.27 – 0.11 | -0.83 | 0.404 | 5036.00 | 0.06 | -0.14 – 0.26 | 0.57 | 0.567 | 5036.00 | 0.17 | -0.09 – 0.43 | 1.25 | 0.210 | 5036.00 | -0.03 | -0.24 – 0.18 | -0.32 | 0.747 | 5036.00 |

| | | | | | | | | | | | | | | | | | | | | |
|---|---|---|---|---|---|---|---|---|---|---|---|---|---|---|---|---|---|---|---|---|
| Valence × Arousal | 0.00 | -0.02 – 0.02 | 0.18 | 0.858 | 5036.00 | -0.06 | -0.08 – -0.04 | -6.08 | **<0.001** | 5036.00 | 0.01 | -0.01 – 0.03 | 0.92 | 0.359 | 5036.00 | 0.04 | 0.01 – 0.06 | 3.04 | **0.002** | 5036.00 |
| TAS DIF × Valence | 0.01 | -0.02 – 0.04 | 0.57 | 0.571 | 5036.00 | -0.02 | -0.05 – 0.01 | -1.29 | 0.196 | 5036.00 | 0.02 | -0.01 – 0.06 | 1.23 | 0.219 | 5036.00 | 0.03 | -0.01 – 0.07 | 1.56 | 0.119 | 5036.00 |
| TAS DIF × Arousal | -0.02 | -0.05 – 0.01 | -1.50 | 0.134 | 5036.00 | 0.01 | -0.02 – 0.04 | 0.80 | 0.422 | 5036.00 | -0.01 | -0.04 – 0.03 | -0.34 | 0.732 | 5036.00 | 0.01 | -0.03 – 0.04 | 0.49 | 0.622 | 5036.00 |
| TAS DIF × Stress | 0.01 | -0.02 – 0.05 | 0.71 | 0.476 | 5036.00 | -0.00 | -0.04 – 0.03 | -0.14 | 0.887 | 5036.00 | -0.01 | -0.04 – 0.03 | -0.47 | 0.640 | 5036.00 | 0.02 | -0.02 – 0.05 | 0.83 | 0.407 | 5036.00 |
| TAS DDF × Valence | -0.03 | -0.07 – 0.00 | -1.80 | 0.072 | 5036.00 | 0.01 | -0.02 – 0.04 | 0.57 | 0.572 | 5036.00 | -0.00 | -0.04 – 0.03 | -0.15 | 0.880 | 5036.00 | -0.01 | -0.04 – 0.03 | -0.36 | 0.716 | 5036.00 |
| TAS DDF × Arousal | -0.00 | -0.04 – 0.03 | -0.31 | 0.758 | 5036.00 | -0.01 | -0.04 – 0.02 | -0.39 | 0.694 | 5036.00 | -0.01 | -0.04 – 0.02 | -0.79 | 0.432 | 5036.00 | -0.01 | -0.05 – 0.02 | -0.78 | 0.436 | 5036.00 |
| TAS DDF × Stress | -0.01 | -0.04 – 0.03 | -0.42 | 0.678 | 5036.00 | -0.02 | -0.05 – 0.02 | -1.08 | 0.282 | 5036.00 | 0.00 | -0.03 – 0.04 | 0.05 | 0.959 | 5036.00 | 0.02 | -0.02 – 0.05 | 0.81 | 0.420 | 5036.00 |
| TAS EOT × Valence | 0.01 | -0.01 – 0.04 | 0.81 | 0.420 | 5036.00 | 0.03 | 0.01 – 0.06 | 2.62 | **0.009** | 5036.00 | 0.03 | 0.01 – 0.06 | 2.48 | 0.013 | 5036.00 | -0.04 | -0.06 – -0.01 | -2.52 | **0.012** | 5036.00 |
| TAS EOT × Arousal | 0.00 | -0.02 – 0.03 | 0.24 | 0.808 | 5036.00 | -0.01 | -0.04 – 0.02 | -0.73 | 0.463 | 5036.00 | -0.01 | -0.04 – 0.02 | -0.84 | 0.403 | 5036.00 | 0.04 | 0.01 – 0.07 | 2.89 | **0.004** | 5036.00 |
| TAS EOT × Stress | -0.00 | -0.03 – 0.03 | -0.05 | 0.958 | 5036.00 | 0.01 | -0.01 – 0.04 | 0.98 | 0.329 | 5036.00 | 0.02 | -0.01 – 0.04 | 1.20 | 0.228 | 5036.00 | -0.05 | -0.08 – -0.02 | -3.17 | **0.002** | 5036.00 |
| (TAS DIF × Valence) × Arousal | 0.03 | 0.01 – 0.06 | 2.67 | **0.008** | 5036.00 | 0.01 | -0.02 – 0.03 | 0.49 | 0.625 | 5036.00 | 0.02 | -0.01 – 0.04 | 1.24 | 0.215 | 5036.00 | 0.00 | -0.03 – 0.03 | 0.06 | 0.954 | 5036.00 |
| (TAS DDF × Valence) × Arousal | -0.01 | -0.04 – 0.01 | -0.95 | 0.341 | 5036.00 | -0.02 | -0.05 – 0.00 | -1.91 | 0.056 | 5036.00 | 0.01 | -0.02 – 0.03 | 0.71 | 0.479 | 5036.00 | -0.01 | -0.04 – 0.02 | -0.58 | 0.561 | 5036.00 |
| (TAS EOT × Valence) × Arousal | -0.02 | -0.04 – 0.00 | -1.60 | 0.111 | 5036.00 | 0.03 | 0.01 – 0.05 | 2.59 | **0.010** | 5036.00 | -0.01 | -0.03 – 0.01 | -0.74 | 0.458 | 5036.00 | 0.00 | -0.02 – 0.02 | 0.03 | 0.980 | 5036.00 |

**Random Effects**

| | | | | |
|---|---|---|---|---|
| $\sigma^2$ | 0.58 | 0.54 | 0.59 | 0.71 |
| $\tau_{00}$ | 0.14 participant_code | 0.16 participant_code | 0.28 participant_code | 0.17 participant_code |
| N | 190 participant_code | 190 participant_code | 190 participant_code | 190 participant_code |
| Observations | 5063 | 5063 | 5063 | 5063 |
| Marginal $R^2$ / Conditional $R^2$ | 0.257 / 0.398 | 0.275 / 0.441 | 0.107 / 0.394 | 0.105 / 0.278 |

**Post-hoc Contrasts for PC 1: Future-self focus (TAS-DIF × Valence × Arousal)**

| *Contrast* | *estimate* | *SE* | *df* | *t* | *p.value* | *conf.low* | *conf.high* |
|---|---|---|---|---|---|---|---|
| LowDIF, Low Arousal: High–Low Valence | 1.1914 | 0.3488 | 5037.8787 | 3.4159 | 0.0006 | 0.5076 | 1.8751 |
| HighDIF, Low Arousal: High–Low Valence | 0.0090 | 0.4024 | 5033.3475 | 0.0225 | 0.9821 | -0.7798 | 0.7979 |
| LowDIF, High Arousal: High–Low Valence | -0.1629 | 0.3321 | 5037.3109 | -0.4905 | 0.6238 | -0.8139 | 0.4882 |
| HighDIF, High Arousal: High–Low Valence | 1.6798 | 0.3481 | 5022.2455 | 4.8259 | 0.0000 | 0.9974 | 2.3622 |
| Contrast of Contrasts (Low Arousal): (High–Low Valence) HighDIF vs LowDIF | -0.2571 | 0.1540 | 5036.3963 | -1.6693 | 0.0951 | -0.5590 | 0.0448 |
| Contrast of Contrasts (High Arousal): (High–Low Valence) HighDIF vs LowDIF | 0.4007 | 0.1394 | 5031.7420 | 2.8744 | 0.0041 | 0.1274 | 0.6740 |

## Post-hoc Contrasts for PC 2: Intrusive distraction (TAS-EOT × Valence × Arousal)

| *Contrast* | *estimate* | *SE* | *df* | *t* | *p.value* | *conf.low* | *conf.high* |
|---|---|---|---|---|---|---|---|

| | | | | | | | |
|---|---|---|---|---|---|---|---|
| LowEOT, Low Arousal: High–Low Valence | -0.3273 | 0.3361 | 5037.2785 | -0.9738 | 0.3302 | -0.9862 | 0.3316 |
| HighEOT, Low Arousal: High–Low Valence | -0.9200 | 0.5650 | 5037.7905 | -1.6283 | 0.1035 | -2.0278 | 0.1877 |
| LowEOT, High Arousal: High–Low Valence | -2.8718 | 0.3073 | 5022.7465 | -9.3464 | 0.0000 | -3.4742 | -2.2694 |
| HighEOT, High Arousal: High–Low Valence | 0.2625 | 0.5283 | 5031.1629 | 0.4970 | 0.6192 | -0.7731 | 1.2981 |
| Contrast of Contrasts (Low Arousal): (High–Low Valence) HighEOT vs LowEOT | -0.1289 | 0.1883 | 5037.9230 | -0.6846 | 0.4936 | -0.4980 | 0.2402 |
| Contrast of Contrasts (High Arousal): (High–Low Valence) HighEOT vs LowEOT | 0.6816 | 0.1746 | 5028.5617 | 3.9031 | 0.0001 | 0.3392 | 1.0239 |

## Post-hoc Contrasts for PC4: Task-focus (TAS-EOT × Valence, TAS-EOT × Arousal, TAS-EOT × Stress)

| *Contrast* | *estimate* | *SE* | *df* | *t* | *p.value* | *conf.low* | *conf.high* |
|---|---|---|---|---|---|---|---|
| LowEOT: High–Low Valence | 1.4418 | 0.1935 | 5034.7175 | 7.4495 | 0.0000 | 1.0624 | 1.8213 |
| HighEOT: High–Low Valence | 0.2095 | 0.3185 | 5035.6356 | 0.6579 | 0.5106 | -0.4149 | 0.8339 |
| LowEOT: High–Low Arousal | 0.0098 | 0.1775 | 5037.1100 | 0.0553 | 0.9559 | -0.3381 | 0.3578 |
| HighEOT: High–Low Arousal | 1.3658 | 0.3085 | 5037.9978 | 4.4267 | 0.0000 | 0.7609 | 1.9706 |
| LowEOT: High–Low Stress | 1.2022 | 0.1855 | 5037.7946 | 6.4825 | 0.0000 | 0.8387 | 1.5658 |
| HighEOT: High–Low Stress | -0.2723 | 0.2991 | 5036.1889 | -0.9105 | 0.3626 | -0.8585 | 0.3140 |
| Contrast of contrasts: (High–Low Valence) High EOT vs LowEOT | -1.2323 | 0.4896 | 5034.5602 | -2.5168 | 0.0119 | -2.1922 | -0.2724 |
| Contrast of contrasts: (High–Low Arousal) High EOT vs LowEOT | 1.3559 | 0.4687 | 5037.7180 | 2.8930 | 0.0038 | 0.4371 | 2.2748 |

| | | | | | | | |
|---|---|---|---|---|---|---|---|
| E Contrast of contrasts: (High–Low Stress) High EOT vs LowEOT | -1.4745 | 0.4645 | 5037.4658 | -3.1743 | 0.0015 | -2.3852 | -0.5639 |

**Supplementary Table 11.** ANOVA outputs and LMM summary tables for the interaction effects of TAS-20 total and Social Environment on 4 thought dimensions

1: Future-self

| *Row* | *Sum Sq* | *Mean Sq* | *NumDF* | *DenDF* | *F value* | *Pr(>F)* |
|---|---|---|---|---|---|---|
| TAS_total | 4.644 | 4.644 | 1 | 185.258 | 6.460 | 0.012 |
| Environment | 32.109 | 32.109 | 1 | 5024.885 | 44.665 | 0.000 |
| DASS_total | 7.474 | 7.474 | 1 | 185.270 | 10.397 | 0.001 |
| Age | 12.649 | 12.649 | 1 | 185.519 | 17.596 | 0.000 |
| Gender | 0.528 | 0.264 | 2 | 183.690 | 0.367 | 0.693 |
| TAS_total:Environment | 0.000 | 0.000 | 1 | 5035.686 | 0.001 | 0.980 |

2: Intrusive distraction

| *Row* | *Sum Sq* | *Mean Sq* | *NumDF* | *DenDF* | *F value* | *Pr(>F)* |
|---|---|---|---|---|---|---|
| TAS_total | 2.026 | 2.026 | 1 | 184.264 | 2.969 | 0.087 |
| Environment | 19.703 | 19.703 | 1 | 5016.574 | 28.880 | 0.000 |
| DASS_total | 25.850 | 25.850 | 1 | 184.288 | 37.890 | 0.000 |
| Age | 0.771 | 0.771 | 1 | 184.503 | 1.130 | 0.289 |
| Gender | 4.299 | 2.150 | 2 | 182.836 | 3.151 | 0.045 |
| TAS_total:Environment | 0.122 | 0.122 | 1 | 5027.970 | 0.179 | 0.672 |

3: Sensory engagement

| *Row* | *Sum Sq* | *Mean Sq* | *NumDF* | *DenDF* | *F value* | *Pr(>F)* |
|---|---|---|---|---|---|---|
| TAS_total | 0.023 | 0.023 | 1 | 184.464 | 0.036 | 0.850 |
| Environment | 27.774 | 27.774 | 1 | 4983.674 | 42.845 | 0.000 |
| DASS_total | 1.933 | 1.933 | 1 | 184.510 | 2.982 | 0.086 |
| Age | 0.018 | 0.018 | 1 | 184.633 | 0.028 | 0.868 |
| Gender | 1.310 | 0.655 | 2 | 183.463 | 1.011 | 0.366 |
| TAS_total:Environment | 4.602 | 4.602 | 1 | 4994.839 | 7.099 | 0.008 |

## 4: Task-focus

| *Row* | *Sum Sq* | *Mean Sq* | *NumDF* | *DenDF* | *F value* | *Pr(>F)* |
|---|---|---|---|---|---|---|
| TAS_total | 0.001 | 0.001 | 1 | 185.341 | 0.001 | 0.976 |
| Environment | 28.231 | 28.231 | 1 | 5031.364 | 37.149 | 0.000 |
| DASS_total | 0.129 | 0.129 | 1 | 185.340 | 0.169 | 0.681 |
| Age | 5.509 | 5.509 | 1 | 185.621 | 7.249 | 0.008 |
| Gender | 0.002 | 0.001 | 2 | 183.654 | 0.001 | 0.999 |
| TAS_total:Environment | 0.309 | 0.309 | 1 | 5041.438 | 0.406 | 0.524 |

| | **Future-self** | | | | | **Intrusive distraction** | | | | | **Sensory engagement** | | | | | **Task-focus** | | | | |
|---|---|---|---|---|---|---|---|---|---|---|---|---|---|---|---|---|---|---|---|---|
| *Predictors* | *Estimates* | *CI* | *Statistic* | *p* | *df* | *Estimates* | *CI* | *Statistic* | *p* | *df* | *Estimates* | *CI* | *Statistic* | *p* | *df* | *Estimates* | *CI* | *Statistic* | *p* | *df* |
| (Intercept) | -0.00 | -0.21 – 0.20 | -0.03 | 0.974 | 5053.00 | 0.12 | -0.09 – 0.33 | 1.14 | 0.256 | 5053.00 | -0.10 | -0.34 – 0.15 | -0.78 | 0.436 | 5053.00 | -0.03 | -0.23 – 0.18 | -0.26 | 0.794 | 5053.00 |

| | | | | | | | | | | | | | | | | | | | | |
|---|---|---|---|---|---|---|---|---|---|---|---|---|---|---|---|---|---|---|---|---|
| TAS total | -0.12 | -0.21 – -0.03 | -2.54 | **0.011** | 5053.00 | -0.08 | -0.18 – 0.01 | -1.72 | 0.085 | 5053.00 | -0.01 | -0.12 – 0.10 | -0.19 | 0.849 | 5053.00 | -0.00 | -0.09 – 0.09 | -0.03 | 0.976 | 5053.00 |
| Environment1 | 0.09 | 0.06 – 0.11 | 6.68 | **<0.001** | 5053.00 | -0.07 | -0.09 – -0.04 | -5.37 | **<0.001** | 5053.00 | 0.08 | 0.06 – 0.11 | 6.55 | **<0.001** | 5053.00 | 0.08 | 0.06 – 0.11 | 6.09 | **<0.001** | 5053.00 |
| DASS total | 0.15 | 0.06 – 0.24 | 3.22 | **0.001** | 5053.00 | 0.29 | 0.19 – 0.38 | 6.16 | **<0.001** | 5053.00 | 0.09 | -0.01 – 0.20 | 1.73 | 0.084 | 5053.00 | -0.02 | -0.11 – 0.07 | -0.41 | 0.681 | 5053.00 |
| Age | 0.16 | 0.09 – 0.24 | 4.19 | **<0.001** | 5053.00 | 0.04 | -0.04 – 0.12 | 1.06 | 0.288 | 5053.00 | 0.01 | -0.08 – 0.10 | 0.17 | 0.868 | 5053.00 | 0.10 | 0.03 – 0.18 | 2.69 | **0.007** | 5053.00 |
| Gender1 | -0.02 | -0.23 – 0.19 | -0.16 | 0.872 | 5053.00 | -0.14 | -0.36 – 0.07 | -1.30 | 0.194 | 5053.00 | 0.08 | -0.17 – 0.33 | 0.60 | 0.551 | 5053.00 | -0.00 | -0.21 – 0.21 | -0.00 | 1.000 | 5053.00 |
| Gender2 | 0.07 | -0.16 – 0.30 | 0.60 | 0.551 | 5053.00 | 0.12 | -0.12 – 0.36 | 1.01 | 0.312 | 5053.00 | 0.20 | -0.08 – 0.48 | 1.40 | 0.162 | 5053.00 | -0.00 | -0.24 – 0.23 | -0.04 | 0.968 | 5053.00 |
| TAS total × Environment1 | 0.00 | -0.03 – 0.03 | 0.03 | 0.980 | 5053.00 | -0.01 | -0.03 – 0.02 | -0.42 | 0.672 | 5053.00 | 0.03 | 0.01 – 0.06 | 2.66 | **0.008** | 5053.00 | 0.01 | -0.02 – 0.04 | 0.64 | 0.524 | 5053.00 |
| **Random Effects** | | | | | | | | | | | | | | | | | | | | |
| $\sigma^2$ | 0.72 | | | | | 0.68 | | | | | 0.65 | | | | | 0.76 | | | | |
| $\tau_{00}$ | 0.23 participant_code | | | | | 0.24 participant_code | | | | | 0.34 participant_code | | | | | 0.22 participant_code | | | | |
| N | 190 participant_code | | | | | 190 participant_code | | | | | 190 participant_code | | | | | 190 participant_code | | | | |
| Observations | 5063 | | | | | 5063 | | | | | 5063 | | | | | 5063 | | | | |
| Marginal $R^2$ / Conditional $R^2$ | 0.057 / 0.287 | | | | | 0.072 / 0.318 | | | | | 0.019 / 0.359 | | | | | 0.017 / 0.242 | | | | |

## Post-hoc Contrasts for PC 3: Sensory Engagement (TAS-total × Social Environment)

| *Contrast* | *estimate* | *SE* | *df* | *t* | *p.value* | *conf.low* | *conf.high* |
|---|---|---|---|---|---|---|---|
| LowTAS: Social – Alone | 0.0044 | 0.0659 | 5008.4113 | 0.0662 | 0.9473 | -0.1248 | 0.1335 |

| | | | | | | | |
|---|---|---|---|---|---|---|---|
| HighTAS: Social – Alone | 0.3321 | 0.0671 | 4976.0529 | 4.9523 | 0.0000 | 0.2006 | 0.4636 |
| Contrast of contrasts: (Social–Alone) HighTAS vs LowTAS | 0.3277 | 0.1230 | 4994.8393 | 2.6645 | 0.0077 | 0.0866 | 0.5689 |

**Supplementary Table 12.** ANOVA outputs and LMM summary tables (with post-hoc contrasts) for the interaction effects of TAS-20 subscales and Social Environment on 4 thought dimensions

1: Future-self

| *Row* | *Sum Sq* | *Mean Sq* | *NumDF* | *DenDF* | *F value* | *Pr(>F)* |
|---|---|---|---|---|---|---|
| TAS_DIF | 0.787 | 0.787 | 1 | 183.523 | 1.096 | 0.296 |
| TAS_DDF | 3.486 | 3.486 | 1 | 184.133 | 4.853 | 0.029 |
| TAS_EOT | 0.081 | 0.081 | 1 | 183.875 | 0.112 | 0.738 |
| Environment | 32.018 | 32.018 | 1 | 5023.986 | 44.575 | 0.000 |
| DASS_total | 7.997 | 7.997 | 1 | 183.599 | 11.133 | 0.001 |
| Age | 13.137 | 13.137 | 1 | 184.251 | 18.290 | 0.000 |
| Gender | 0.540 | 0.270 | 2 | 181.704 | 0.376 | 0.687 |
| TAS_DIF:Environment | 3.499 | 3.499 | 1 | 5018.852 | 4.871 | 0.027 |
| TAS_DDF:Environment | 3.544 | 3.544 | 1 | 5009.376 | 4.934 | 0.026 |
| TAS_EOT:Environment | 0.000 | 0.000 | 1 | 5024.878 | 0.000 | 0.999 |

2: Intrusive distraction

| *Row* | *Sum Sq* | *Mean Sq* | *NumDF* | *DenDF* | *F value* | *Pr(>F)* |
|---|---|---|---|---|---|---|
| TAS_DIF | 0.060 | 0.060 | 1 | 182.030 | 0.088 | 0.768 |
| TAS_DDF | 3.434 | 3.434 | 1 | 182.589 | 5.040 | 0.026 |
| TAS_EOT | 0.299 | 0.299 | 1 | 182.352 | 0.438 | 0.509 |
| Environment | 19.019 | 19.019 | 1 | 5016.810 | 27.909 | 0.000 |

| | | | | | | |
|---|---|---|---|---|---|---|
| DASS_total | 25.325 | 25.325 | 1 | 182.104 | 37.163 | 0.000 |
| Age | 0.818 | 0.818 | 1 | 182.695 | 1.201 | 0.275 |
| Gender | 4.447 | 2.223 | 2 | 180.352 | 3.263 | 0.041 |
| TAS_DIF:Environment | 1.823 | 1.823 | 1 | 5011.440 | 2.675 | 0.102 |
| TAS_DDF:Environment | 0.006 | 0.006 | 1 | 5001.896 | 0.008 | 0.928 |
| TAS_EOT:Environment | 3.857 | 3.857 | 1 | 5017.658 | 5.660 | 0.017 |

3: Sensory engagement

| *Row* | *Sum Sq* | *Mean Sq* | *NumDF* | *DenDF* | *F value* | *Pr(>F)* |
|---|---|---|---|---|---|---|
| TAS_DIF | 0.399 | 0.399 | 1 | 182.419 | 0.616 | 0.434 |
| TAS_DDF | 0.854 | 0.854 | 1 | 182.799 | 1.317 | 0.253 |
| TAS_EOT | 0.025 | 0.025 | 1 | 182.636 | 0.039 | 0.844 |
| Environment | 27.661 | 27.661 | 1 | 4982.362 | 42.653 | 0.000 |
| DASS_total | 1.462 | 1.462 | 1 | 182.481 | 2.254 | 0.135 |
| Age | 0.008 | 0.008 | 1 | 182.865 | 0.012 | 0.914 |
| Gender | 1.781 | 0.891 | 2 | 181.248 | 1.373 | 0.256 |
| TAS_DIF:Environment | 0.910 | 0.910 | 1 | 4977.116 | 1.404 | 0.236 |
| TAS_DDF:Environment | 0.844 | 0.844 | 1 | 4968.874 | 1.302 | 0.254 |
| TAS_EOT:Environment | 0.428 | 0.428 | 1 | 4982.858 | 0.659 | 0.417 |

4: Task-focus

| *Row* | *Sum Sq* | *Mean Sq* | *NumDF* | *DenDF* | *F value* | *Pr(>F)* |
|---|---|---|---|---|---|---|
| TAS_DIF | 1.320 | 1.320 | 1 | 183.526 | 1.738 | 0.189 |
| TAS_DDF | 1.891 | 1.891 | 1 | 184.180 | 2.490 | 0.116 |
| TAS_EOT | 0.105 | 0.105 | 1 | 183.905 | 0.138 | 0.710 |
| Environment | 27.675 | 27.675 | 1 | 5029.792 | 36.437 | 0.000 |
| DASS_total | 0.286 | 0.286 | 1 | 183.603 | 0.376 | 0.540 |
| Age | 5.071 | 5.071 | 1 | 184.309 | 6.676 | 0.011 |
| Gender | 0.117 | 0.059 | 2 | 181.587 | 0.077 | 0.926 |
| TAS_DIF:Environment | 0.553 | 0.553 | 1 | 5024.918 | 0.728 | 0.394 |
| TAS_DDF:Environment | 3.269 | 3.269 | 1 | 5015.557 | 4.303 | 0.038 |
| TAS_EOT:Environment | 0.828 | 0.828 | 1 | 5030.709 | 1.090 | 0.296 |

| | **Future-self** | | | | | **Intrusive distraction** | | | | | **Sensory engagement** | | | | | **Task-focus** | | | | |
|---|---|---|---|---|---|---|---|---|---|---|---|---|---|---|---|---|---|---|---|---|
| *Predictors* | *Estimates* | *CI* | *Statistic* | *p* | *df* | *Estimates* | *CI* | *Statistic* | *p* | *df* | *Estimates* | *CI* | *Statistic* | *p* | *df* | *Estimates* | *CI* | *Statistic* | *p* | *df* |
| (Intercept) | -0.01 | -0.21 – 0.20 | -0.09 | 0.928 | 5049.00 | 0.11 | -0.10 – 0.32 | 1.06 | 0.290 | 5049.00 | -0.11 | -0.35 – 0.14 | -0.85 | 0.398 | 5049.00 | -0.04 | -0.24 – 0.17 | -0.35 | 0.724 | 5049.00 |
| TAS DIF | -0.06 | -0.16 – 0.05 | -1.05 | 0.295 | 5049.00 | -0.02 | -0.12 – 0.09 | -0.30 | 0.767 | 5049.00 | 0.05 | -0.07 – 0.17 | 0.78 | 0.433 | 5049.00 | 0.07 | -0.03 – 0.17 | 1.32 | 0.187 | 5049.00 |
| TAS DDF | -0.10 | -0.19 – -0.01 | -2.20 | 0.028 | 5049.00 | -0.11 | -0.20 – -0.01 | -2.24 | **0.025** | 5049.00 | -0.06 | -0.17 – 0.05 | -1.15 | 0.251 | 5049.00 | -0.07 | -0.16 – 0.02 | -1.58 | 0.115 | 5049.00 |
| TAS EOT | 0.01 | -0.06 – 0.09 | 0.33 | 0.738 | 5049.00 | 0.03 | -0.05 – 0.10 | 0.66 | 0.508 | 5049.00 | 0.01 | -0.08 – 0.10 | 0.20 | 0.844 | 5049.00 | 0.01 | -0.06 – 0.09 | 0.37 | 0.710 | 5049.00 |

| | | | | | | | | | | | | | | | | | | | | |
|---|---|---|---|---|---|---|---|---|---|---|---|---|---|---|---|---|---|---|---|---|
| Environment1 | 0.09 | 0.06 – 0.11 | 6.68 | **<0.001** | 5049.00 | -0.07 | -0.09 – -0.04 | -5.28 | **<0.001** | 5049.00 | 0.08 | 0.06 – 0.11 | 6.53 | **<0.001** | 5049.00 | 0.08 | 0.06 – 0.11 | 6.04 | **<0.001** | 5049.00 |
| DASS total | 0.16 | 0.07 – 0.25 | 3.34 | **0.001** | 5049.00 | 0.29 | 0.20 – 0.39 | 6.10 | **<0.001** | 5049.00 | 0.09 | -0.03 – 0.20 | 1.50 | 0.133 | 5049.00 | -0.03 | -0.12 – 0.06 | -0.61 | 0.540 | 5049.00 |
| Age | 0.17 | 0.09 – 0.24 | 4.28 | **<0.001** | 5049.00 | 0.04 | -0.03 – 0.12 | 1.10 | 0.273 | 5049.00 | 0.01 | -0.09 – 0.10 | 0.11 | 0.914 | 5049.00 | 0.10 | 0.02 – 0.18 | 2.58 | **0.010** | 5049.00 |
| Gender1 | -0.01 | -0.22 – 0.20 | -0.08 | 0.937 | 5049.00 | -0.13 | -0.34 – 0.08 | -1.21 | 0.226 | 5049.00 | 0.08 | -0.17 – 0.33 | 0.64 | 0.523 | 5049.00 | 0.01 | -0.20 – 0.21 | 0.06 | 0.955 | 5049.00 |
| Gender2 | 0.08 | -0.16 – 0.32 | 0.67 | 0.502 | 5049.00 | 0.15 | -0.10 – 0.39 | 1.18 | 0.236 | 5049.00 | 0.24 | -0.05 – 0.53 | 1.62 | 0.104 | 5049.00 | 0.04 | -0.20 – 0.28 | 0.35 | 0.724 | 5049.00 |
| TAS DIF × Environment1 | -0.03 | -0.07 – -0.00 | -2.21 | 0.027 | 5049.00 | -0.03 | -0.06 – 0.01 | -1.64 | 0.102 | 5049.00 | 0.02 | -0.01 – 0.05 | 1.18 | 0.236 | 5049.00 | -0.01 | -0.05 – 0.02 | -0.85 | 0.394 | 5049.00 |
| TAS DDF × Environment1 | 0.03 | 0.00 – 0.07 | 2.22 | 0.026 | 5049.00 | -0.00 | -0.03 – 0.03 | -0.09 | 0.928 | 5049.00 | 0.02 | -0.01 – 0.05 | 1.14 | 0.254 | 5049.00 | 0.03 | 0.00 – 0.07 | 2.07 | 0.038 | 5049.00 |
| TAS EOT × Environment1 | 0.00 | -0.03 – 0.03 | 0.00 | 0.999 | 5049.00 | 0.03 | 0.01 – 0.06 | 2.38 | 0.017 | 5049.00 | 0.01 | -0.01 – 0.04 | 0.81 | 0.417 | 5049.00 | -0.01 | -0.04 – 0.01 | -1.04 | 0.296 | 5049.00 |
| **Random Effects** | | | | | | | | | | | | | | | | | | | | |
| $\sigma^2$ | 0.72 | | | | | 0.68 | | | | | 0.65 | | | | | 0.76 | | | | |
| $\tau_{00}$ | 0.23 participant_code | | | | | 0.24 participant_code | | | | | 0.35 participant_code | | | | | 0.22 participant_code | | | | |
| N | 190 participant_code | | | | | 190 participant_code | | | | | 190 participant_code | | | | | 190 participant_code | | | | |
| Observations | 5063 | | | | | 5063 | | | | | 5063 | | | | | 5063 | | | | |
| Marginal $R^2$ / Conditional $R^2$ | 0.062 / 0.289 | | | | | 0.079 / 0.319 | | | | | 0.022 / 0.361 | | | | | 0.022 / 0.245 | | | | |

**Supplementary Table 13.** Additional analysis using TAS-total score to predict affective states. LMM summary tables for the main effects of TAS-20 total score on 3 affective dimensions

| | Valence | | | | | Arousal | | | | | Stress | | | | |
|---|---|---|---|---|---|---|---|---|---|---|---|---|---|---|---|
| *Predictors* | *Estimates* | *CI* | *Statistic* | *p* | *df* | *Estimates* | *CI* | *Statistic* | *p* | *df* | *Estimates* | *CI* | *Statistic* | *p* | *df* |
| (Intercept) | 5.59 | 5.15 – 6.02 | 25.08 | **<0.001** | 5055.00 | 4.23 | 3.73 – 4.72 | 16.62 | **<0.001** | 5055.00 | 2.92 | 2.47 – 3.37 | 12.73 | **<0.001** | 5055.00 |
| TAS total | 0.02 | -0.18 – 0.22 | 0.21 | 0.833 | 5055.00 | -0.22 | -0.44 – 0.01 | -1.91 | 0.056 | 5055.00 | -0.17 | -0.38 – 0.03 | -1.68 | 0.094 | 5055.00 |
| DASS total | -0.39 | -0.58 – -0.20 | -4.03 | **<0.001** | 5055.00 | 0.29 | 0.07 – 0.50 | 2.58 | **0.010** | 5055.00 | 0.72 | 0.52 – 0.91 | 7.20 | **<0.001** | 5055.00 |
| Age | 0.05 | -0.11 – 0.21 | 0.61 | 0.543 | 5055.00 | 0.24 | 0.06 – 0.43 | 2.57 | **0.010** | 5055.00 | 0.05 | -0.12 – 0.22 | 0.58 | 0.560 | 5055.00 |
| Gender1 | 0.05 | -0.39 – 0.50 | 0.24 | 0.810 | 5055.00 | 0.37 | -0.15 – 0.88 | 1.40 | 0.161 | 5055.00 | -0.02 | -0.48 – 0.44 | -0.08 | 0.940 | 5055.00 |
| Gender2 | 0.05 | -0.45 – 0.54 | 0.19 | 0.851 | 5055.00 | 0.80 | 0.24 – 1.37 | 2.77 | **0.006** | 5055.00 | 0.43 | -0.09 – 0.94 | 1.63 | 0.103 | 5055.00 |
| **Random Effects** | | | | | | | | | | | | | | | |
| $\sigma^2$ | 3.60 | | | | | 4.27 | | | | | 4.33 | | | | |
| $\tau_{00}$ | 1.03 $_{participant\_code}$ | | | | | 1.36 $_{participant\_code}$ | | | | | 1.08 $_{participant\_code}$ | | | | |
| N | 190 $_{participant\_code}$ | | | | | 190 $_{participant\_code}$ | | | | | 190 $_{participant\_code}$ | | | | |
| Observations | 5063 | | | | | 5063 | | | | | 5063 | | | | |
| Marginal $R^2$ / Conditional $R^2$ | 0.032 / 0.248 | | | | | 0.030 / 0.265 | | | | | 0.070 / 0.256 | | | | |

**Supplementary Table 14.** Additional analysis using TAS-subscale scores to predict affective states. LMM summary tables for the main effects of 3 TAS-20 subscales on 3 affective dimensions

| | **Valence** | | | | | **Arousal** | | | | | **Stress** | | | | |
|---|---|---|---|---|---|---|---|---|---|---|---|---|---|---|---|
| *Predictors* | *Estimates* | *CI* | *Statistic* | *p* | *df* | *Estimates* | *CI* | *Statistic* | *p* | *df* | *Estimates* | *CI* | *Statistic* | *p* | *df* |
| (Intercept) | 5.60 | 5.16 – 6.04 | 25.02 | **<0.001** | 5053.00 | 4.20 | 3.70 – 4.70 | 16.53 | **<0.001** | 5053.00 | 2.89 | 2.45 – 3.33 | 12.82 | **<0.001** | 5053.00 |
| TAS DIF | -0.00 | -0.22 – 0.22 | -0.02 | 0.980 | 5053.00 | -0.06 | -0.31 – 0.20 | -0.43 | 0.666 | 5053.00 | -0.08 | -0.31 – 0.14 | -0.71 | 0.478 | 5053.00 |
| TAS DDF | 0.07 | -0.12 – 0.27 | 0.75 | 0.455 | 5053.00 | -0.24 | -0.46 – -0.02 | -2.12 | 0.034 | 5053.00 | -0.25 | -0.44 – -0.05 | -2.43 | **0.015** | 5053.00 |
| TAS EOT | -0.07 | -0.23 – 0.10 | -0.79 | 0.428 | 5053.00 | 0.04 | -0.15 – 0.23 | 0.43 | 0.671 | 5053.00 | 0.16 | -0.01 – 0.32 | 1.86 | 0.062 | 5053.00 |
| DASS total | -0.40 | -0.60 – -0.20 | -3.98 | **<0.001** | 5053.00 | 0.30 | 0.07 – 0.52 | 2.58 | **0.010** | 5053.00 | 0.77 | 0.56 – 0.97 | 7.47 | **<0.001** | 5053.00 |
| Age | 0.04 | -0.12 – 0.21 | 0.51 | 0.613 | 5053.00 | 0.25 | 0.06 – 0.44 | 2.64 | **0.008** | 5053.00 | 0.07 | -0.09 – 0.24 | 0.89 | 0.375 | 5053.00 |
| Gender1 | 0.04 | -0.41 – 0.49 | 0.18 | 0.857 | 5053.00 | 0.39 | -0.12 – 0.90 | 1.50 | 0.134 | 5053.00 | 0.02 | -0.43 – 0.47 | 0.09 | 0.930 | 5053.00 |
| Gender2 | 0.03 | -0.49 – 0.54 | 0.11 | 0.914 | 5053.00 | 0.87 | 0.28 – 1.45 | 2.91 | **0.004** | 5053.00 | 0.47 | -0.05 – 0.98 | 1.76 | 0.078 | 5053.00 |
| **Random Effects** | | | | | | | | | | | | | | | |
| $\sigma^2$ | 3.60 | | | | | 4.27 | | | | | 4.33 | | | | |
| $\tau_{00}$ | 1.04 $_{participant\_code}$ | | | | | 1.36 $_{participant\_code}$ | | | | | 1.03 $_{participant\_code}$ | | | | |
| N | 190 $_{participant\_code}$ | | | | | 190 $_{participant\_code}$ | | | | | 190 $_{participant\_code}$ | | | | |
| Observations | 5063 | | | | | 5063 | | | | | 5063 | | | | |
| Marginal $R^2$ / Conditional $R^2$ | 0.033 / 0.250 | | | | | 0.034 / 0.267 | | | | | 0.080 / 0.257 | | | | |

**Supplementary Table 15.** Additional analysis controlling for time (probe position). LMM summary tables for the main and interaction effects of affective states (valence, arousal, and stress) on 4 thought dimensions

| | **Future-self focus** | | | | | **Intrusive distraction** | | | | | **Sensory Engagement** | | | | | **Task-focus** | | | | |
|---|---|---|---|---|---|---|---|---|---|---|---|---|---|---|---|---|---|---|---|---|
| *Predictors* | *Estimates* | *CI* | *Statistic* | *p* | *df* | *Estimates* | *CI* | *Statistic* | *p* | *df* | *Estimates* | *CI* | *Statistic* | *p* | *df* | *Estimates* | *CI* | *Statistic* | *p* | *df* |
| (Intercept) | 0.16 | -0.00 – 0.33 | 1.91 | 0.057 | 5051.00 | 0.18 | 0.00 – 0.36 | 1.97 | 0.049 | 5051.00 | -0.04 | -0.27 – 0.19 | -0.34 | 0.734 | 5051.00 | -0.07 | -0.25 – 0.12 | -0.71 | 0.476 | 5051.00 |
| Valence | 0.15 | 0.12 – 0.18 | 10.46 | **<0.001** | 5051.00 | -0.24 | -0.27 – -0.21 | -17.30 | **<0.001** | 5051.00 | 0.20 | 0.17 – 0.23 | 13.76 | **<0.001** | 5051.00 | 0.21 | 0.18 – 0.25 | 13.73 | **<0.001** | 5051.00 |
| Arousal | 0.30 | 0.28 – 0.33 | 22.96 | **<0.001** | 5051.00 | 0.16 | 0.13 – 0.18 | 12.08 | **<0.001** | 5051.00 | 0.17 | 0.15 – 0.20 | 12.89 | **<0.001** | 5051.00 | 0.12 | 0.09 – 0.15 | 8.01 | **<0.001** | 5051.00 |
| Stress | 0.25 | 0.22 – 0.27 | 16.91 | **<0.001** | 5051.00 | 0.22 | 0.19 – 0.25 | 15.53 | **<0.001** | 5051.00 | -0.00 | -0.03 – 0.03 | -0.14 | 0.888 | 5051.00 | 0.16 | 0.13 – 0.19 | 9.91 | **<0.001** | 5051.00 |
| Environment1 | 0.03 | 0.01 – 0.05 | 2.55 | **0.011** | 5051.00 | -0.07 | -0.09 – -0.04 | -5.63 | **<0.001** | 5051.00 | 0.04 | 0.02 – 0.06 | 3.27 | **0.001** | 5051.00 | 0.03 | 0.00 – 0.06 | 2.21 | 0.027 | 5051.00 |
| Age | 0.14 | 0.08 – 0.19 | 4.66 | **<0.001** | 5051.00 | 0.03 | -0.03 – 0.10 | 1.03 | 0.301 | 5051.00 | -0.03 | -0.11 – 0.05 | -0.74 | 0.456 | 5051.00 | 0.08 | 0.01 – 0.14 | 2.42 | 0.016 | 5051.00 |
| Gender1 | -0.07 | -0.24 – 0.09 | -0.86 | 0.391 | 5051.00 | -0.18 | -0.36 – 0.00 | -1.96 | 0.050 | 5051.00 | 0.02 | -0.21 – 0.25 | 0.15 | 0.884 | 5051.00 | -0.02 | -0.20 – 0.17 | -0.16 | 0.871 | 5051.00 |
| Gender2 | -0.10 | -0.28 – 0.08 | -1.08 | 0.278 | 5051.00 | -0.02 | -0.22 – 0.18 | -0.20 | 0.840 | 5051.00 | 0.08 | -0.17 – 0.34 | 0.62 | 0.533 | 5051.00 | -0.07 | -0.27 – 0.13 | -0.67 | 0.500 | 5051.00 |
| time | -0.03 | -0.04 – -0.02 | -4.48 | **<0.001** | 5051.00 | 0.00 | -0.01 – 0.01 | 0.19 | 0.851 | 5051.00 | 0.00 | -0.01 – 0.02 | 0.64 | 0.521 | 5051.00 | 0.02 | 0.01 – 0.03 | 2.78 | **0.006** | 5051.00 |
| Valence × Arousal | 0.01 | -0.01 – 0.03 | 0.86 | 0.392 | 5051.00 | -0.07 | -0.09 – -0.05 | -6.55 | **<0.001** | 5051.00 | 0.01 | -0.01 – 0.03 | 1.20 | 0.230 | 5051.00 | 0.03 | 0.01 – 0.06 | 3.07 | **0.002** | 5051.00 |
| **Random Effects** | | | | | | | | | | | | | | | | | | | | |
| $\sigma^2$ | 0.58 | | | | | 0.54 | | | | | 0.59 | | | | | 0.71 | | | | |

| | | | | |
|---|---|---|---|---|
| $\tau_{00}$ | 0.14 participant_code | 0.18 participant_code | 0.29 participant_code | 0.17 participant_code |
| N | 190 participant_code | 190 participant_code | 190 participant_code | 190 participant_code |
| Observations | 5063 | 5063 | 5063 | 5063 |
| Marginal $R^2$ / Conditional $R^2$ | 0.247 / 0.393 | 0.233 / 0.420 | 0.093 / 0.392 | 0.099 / 0.273 |

**Supplementary Table 16.** Additional analysis controlling for time (probe position). LMM summary tables for the main effect of TAS-20 total score on 4 thought dimensions

| | **Future-self focus** | | | | | **Intrusive distraction** | | | | | **Sensory Engagement** | | | | | **Task-focus** | | | | |
|---|---|---|---|---|---|---|---|---|---|---|---|---|---|---|---|---|---|---|---|---|
| *Predictors* | *Estimates* | *CI* | *Statistic* | *p* | *df* | *Estimates* | *CI* | *Statistic* | *p* | *df* | *Estimates* | *CI* | *Statistic* | *p* | *df* | *Estimates* | *CI* | *Statistic* | *p* | *df* |
| (Intercept) | 0.05 | -0.16 – 0.26 | 0.48 | 0.634 | 5054.00 | 0.14 | -0.08 – 0.35 | 1.23 | 0.219 | 5054.00 | -0.15 | -0.40 – 0.10 | -1.17 | 0.243 | 5054.00 | -0.11 | -0.32 – 0.09 | -1.08 | 0.281 | 5054.00 |
| TAS total | -0.12 | -0.21 – -0.03 | -2.56 | **0.011** | 5054.00 | -0.08 | -0.18 – 0.01 | -1.71 | 0.088 | 5054.00 | -0.01 | -0.12 – 0.10 | -0.12 | 0.906 | 5054.00 | -0.00 | -0.09 – 0.09 | -0.03 | 0.979 | 5054.00 |
| DASS total | 0.15 | 0.06 – 0.24 | 3.37 | **0.001** | 5054.00 | 0.28 | 0.19 – 0.37 | 6.01 | **<0.001** | 5054.00 | 0.10 | -0.00 – 0.21 | 1.91 | 0.056 | 5054.00 | -0.01 | -0.10 – 0.08 | -0.20 | 0.844 | 5054.00 |
| Age | 0.16 | 0.08 – 0.24 | 4.13 | **<0.001** | 5054.00 | 0.05 | -0.03 – 0.12 | 1.15 | 0.250 | 5054.00 | 0.01 | -0.08 – 0.10 | 0.17 | 0.868 | 5054.00 | 0.10 | 0.03 – 0.18 | 2.66 | **0.008** | 5054.00 |
| Gender1 | -0.01 | -0.22 – 0.20 | -0.11 | 0.911 | 5054.00 | -0.14 | -0.36 – 0.07 | -1.28 | 0.199 | 5054.00 | 0.08 | -0.17 – 0.33 | 0.65 | 0.516 | 5054.00 | 0.01 | -0.20 – 0.21 | 0.05 | 0.959 | 5054.00 |
| Gender2 | 0.07 | -0.17 – 0.30 | 0.55 | 0.580 | 5054.00 | 0.13 | -0.11 – 0.37 | 1.03 | 0.303 | 5054.00 | 0.19 | -0.09 – 0.47 | 1.35 | 0.179 | 5054.00 | -0.01 | -0.24 – 0.22 | -0.07 | 0.941 | 5054.00 |
| time | -0.01 | -0.03 – 0.00 | -1.92 | 0.055 | 5054.00 | -0.01 | -0.02 – 0.01 | -0.97 | 0.330 | 5054.00 | 0.02 | 0.01 – 0.03 | 2.85 | **0.004** | 5054.00 | 0.03 | 0.02 – 0.04 | 4.19 | **<0.001** | 5054.00 |
| **Random Effects** | | | | | | | | | | | | | | | | | | | | |
| $\sigma^2$ | 0.72 | | | | | 0.70 | | | | | 0.65 | | | | | 0.76 | | | | |
| $\tau_{00}$ | 0.23 participant_code | | | | | 0.25 participant_code | | | | | 0.34 participant_code | | | | | 0.22 participant_code | | | | |
| N | 190 participant_code | | | | | 190 participant_code | | | | | 190 participant_code | | | | | 190 participant_code | | | | |
| Observations | 5063 | | | | | 5063 | | | | | 5063 | | | | | 5063 | | | | |
| Marginal $R^2$ / Conditional $R^2$ | 0.050 / 0.280 | | | | | 0.068 / 0.312 | | | | | 0.013 / 0.353 | | | | | 0.013 / 0.238 | | | | |

**Supplementary Table 17.** Additional analysis controlling for time (probe position). LMM summary tables for the interaction effects of TAS-20 total score and affective states on 4 thought dimensions

| | **Future-self focus** | | | | | **Intrusive distraction** | | | | | **Sensory Engagement** | | | | | **Task-focus** | | | | |
|---|---|---|---|---|---|---|---|---|---|---|---|---|---|---|---|---|---|---|---|---|
| *Predictors* | *Estimates* | *CI* | *Statistic* | *p* | *df* | *Estimates* | *CI* | *Statistic* | *p* | *df* | *Estimates* | *CI* | *Statistic* | *p* | *df* | *Estimates* | *CI* | *Statistic* | *p* | *df* |
| (Intercept) | 0.15 | -0.02 – 0.32 | 1.77 | 0.076 | 5045.00 | 0.15 | -0.03 – 0.32 | 1.65 | 0.100 | 5045.00 | -0.06 | -0.29 – 0.16 | -0.54 | 0.587 | 5045.00 | -0.06 | -0.25 – 0.12 | -0.66 | 0.509 | 5045.00 |
| TAS total | -0.08 | -0.15 – -0.01 | -2.10 | 0.036 | 5045.00 | -0.05 | -0.13 – 0.03 | -1.23 | 0.217 | 5045.00 | 0.01 | -0.09 – 0.11 | 0.16 | 0.871 | 5045.00 | 0.02 | -0.06 – 0.10 | 0.46 | 0.648 | 5045.00 |
| Valence | 0.15 | 0.12 – 0.18 | 10.51 | **<0.001** | 5045.00 | -0.24 | -0.26 – -0.21 | -17.20 | **<0.001** | 5045.00 | 0.20 | 0.17 – 0.23 | 13.74 | **<0.001** | 5045.00 | 0.21 | 0.18 – 0.25 | 13.68 | **<0.001** | 5045.00 |
| Arousal | 0.31 | 0.28 – 0.33 | 22.94 | **<0.001** | 5045.00 | 0.16 | 0.13 – 0.18 | 12.06 | **<0.001** | 5045.00 | 0.18 | 0.15 – 0.20 | 13.14 | **<0.001** | 5045.00 | 0.12 | 0.09 – 0.14 | 7.88 | **<0.001** | 5045.00 |
| Stress | 0.24 | 0.21 – 0.27 | 16.69 | **<0.001** | 5045.00 | 0.22 | 0.19 – 0.24 | 15.27 | **<0.001** | 5045.00 | -0.01 | -0.03 – 0.02 | -0.34 | 0.735 | 5045.00 | 0.16 | 0.13 – 0.19 | 9.92 | **<0.001** | 5045.00 |
| Environment1 | 0.03 | 0.01 – 0.05 | 2.47 | 0.014 | 5045.00 | -0.07 | -0.09 – -0.04 | -5.75 | **<0.001** | 5045.00 | 0.04 | 0.01 – 0.06 | 3.06 | **0.002** | 5045.00 | 0.03 | 0.00 – 0.06 | 2.28 | 0.022 | 5045.00 |
| DASS total | 0.07 | -0.00 – 0.14 | 1.85 | 0.064 | 5045.00 | 0.15 | 0.08 – 0.23 | 3.98 | **<0.001** | 5045.00 | 0.12 | 0.02 – 0.21 | 2.38 | 0.017 | 5045.00 | -0.03 | -0.11 – 0.05 | -0.72 | 0.471 | 5045.00 |
| Age | 0.12 | 0.06 – 0.18 | 3.90 | **<0.001** | 5045.00 | 0.04 | -0.03 – 0.10 | 1.10 | 0.271 | 5045.00 | -0.01 | -0.09 – 0.07 | -0.28 | 0.778 | 5045.00 | 0.08 | 0.01 – 0.15 | 2.36 | 0.018 | 5045.00 |
| Gender1 | -0.06 | -0.23 – 0.10 | -0.76 | 0.449 | 5045.00 | -0.15 | -0.33 – 0.03 | -1.67 | 0.095 | 5045.00 | 0.04 | -0.19 – 0.27 | 0.34 | 0.731 | 5045.00 | -0.02 | -0.20 – 0.17 | -0.20 | 0.844 | 5045.00 |
| Gender2 | -0.08 | -0.27 – 0.10 | -0.90 | 0.368 | 5045.00 | 0.04 | -0.16 – 0.23 | 0.38 | 0.704 | 5045.00 | 0.13 | -0.12 – 0.38 | 1.04 | 0.300 | 5045.00 | -0.08 | -0.28 – 0.12 | -0.76 | 0.446 | 5045.00 |
| time | -0.03 | -0.04 – -0.02 | -4.43 | **<0.001** | 5045.00 | 0.00 | -0.01 – 0.01 | 0.23 | 0.818 | 5045.00 | 0.00 | -0.01 – 0.02 | 0.64 | 0.521 | 5045.00 | 0.02 | 0.01 – 0.03 | 2.75 | **0.006** | 5045.00 |

| | | | | | | | | | | | | | | | | | | | | |
|---|---|---|---|---|---|---|---|---|---|---|---|---|---|---|---|---|---|---|---|---|
| Valence × Arousal | 0.01 | -0.01 – 0.03 | 0.68 | 0.494 | 5045.00 | -0.06 | -0.08 – -0.05 | -6.47 | **<0.001** | 5045.00 | 0.01 | -0.01 – 0.03 | 1.12 | 0.261 | 5045.00 | 0.04 | 0.01 – 0.06 | 3.15 | **0.002** | 5045.00 |
| TAS total × Valence | -0.01 | -0.04 – 0.02 | -0.65 | 0.513 | 5045.00 | 0.01 | -0.02 – 0.03 | 0.70 | 0.486 | 5045.00 | 0.04 | 0.01 – 0.06 | 2.59 | **0.010** | 5045.00 | -0.00 | -0.03 – 0.03 | -0.25 | 0.805 | 5045.00 |
| TAS total × Arousal | -0.02 | -0.04 – 0.01 | -1.52 | 0.128 | 5045.00 | -0.00 | -0.03 – 0.02 | -0.05 | 0.963 | 5045.00 | -0.02 | -0.04 – 0.01 | -1.44 | 0.151 | 5045.00 | 0.01 | -0.01 – 0.04 | 0.96 | 0.338 | 5045.00 |
| TAS total × Stress | 0.01 | -0.02 – 0.03 | 0.45 | 0.653 | 5045.00 | -0.01 | -0.03 – 0.02 | -0.46 | 0.647 | 5045.00 | 0.00 | -0.02 – 0.03 | 0.30 | 0.762 | 5045.00 | -0.00 | -0.03 – 0.03 | -0.07 | 0.946 | 5045.00 |
| (TAS total × Valence) × Arousal | 0.01 | -0.01 – 0.03 | 0.87 | 0.385 | 5045.00 | 0.00 | -0.02 – 0.02 | 0.04 | 0.968 | 5045.00 | 0.02 | -0.00 – 0.03 | 1.65 | 0.100 | 5045.00 | -0.01 | -0.03 – 0.01 | -0.75 | 0.451 | 5045.00 |
| **Random Effects** | | | | | | | | | | | | | | | | | | | | |
| $\sigma^2$ | 0.58 | | | | | 0.54 | | | | | 0.59 | | | | | 0.71 | | | | |
| $\tau_{00}$ | 0.14 participant_code | | | | | 0.16 participant_code | | | | | 0.28 participant_code | | | | | 0.17 participant_code | | | | |
| N | 190 participant_code | | | | | 190 participant_code | | | | | 190 participant_code | | | | | 190 participant_code | | | | |
| Observations | 5063 | | | | | 5063 | | | | | 5063 | | | | | 5063 | | | | |
| Marginal $R^2$ / Conditional $R^2$ | 0.257 / 0.397 | | | | | 0.269 / 0.436 | | | | | 0.104 / 0.391 | | | | | 0.099 / 0.274 | | | | |

**Supplementary Table 18.** Additional analysis controlling for time (probe position). LMM summary tables for the interaction effects of TAS-20 subscales and affective states on 4 thought dimensions

| | **Future-self focus** | | | | | **Intrusive distraction** | | | | | **Sensory engagement** | | | | | **Task-focus** | | | | |
|---|---|---|---|---|---|---|---|---|---|---|---|---|---|---|---|---|---|---|---|---|
| *Predictors* | *Estimates* | *CI* | *Statistic* | *p* | *df* | *Estimates* | *CI* | *Statistic* | *p* | *df* | *Estimates* | *CI* | *Statistic* | *p* | *df* | *Estimates* | *CI* | *Statistic* | *p* | *df* |
| (Intercept) | 0.15 | -0.02 – 0.32 | 1.77 | 0.077 | 5035.00 | 0.14 | -0.04 – 0.32 | 1.55 | 0.120 | 5035.00 | -0.07 | -0.29 – 0.16 | -0.59 | 0.554 | 5035.00 | -0.07 | -0.25 – 0.12 | -0.71 | 0.475 | 5035.00 |
| TAS DIF | -0.05 | -0.13 – 0.03 | -1.17 | 0.241 | 5035.00 | -0.01 | -0.09 – 0.08 | -0.12 | 0.907 | 5035.00 | 0.06 | -0.06 – 0.17 | 0.98 | 0.325 | 5035.00 | 0.07 | -0.02 – 0.16 | 1.53 | 0.126 | 5035.00 |
| TAS DDF | -0.05 | -0.12 – 0.02 | -1.29 | 0.198 | 5035.00 | -0.06 | -0.14 – 0.02 | -1.51 | 0.132 | 5035.00 | -0.05 | -0.15 – 0.04 | -1.07 | 0.283 | 5035.00 | -0.05 | -0.13 – 0.03 | -1.21 | 0.226 | 5035.00 |
| TAS EOT | -0.00 | -0.06 – 0.06 | -0.02 | 0.985 | 5035.00 | 0.01 | -0.06 – 0.07 | 0.26 | 0.796 | 5035.00 | 0.02 | -0.06 – 0.10 | 0.50 | 0.620 | 5035.00 | 0.01 | -0.06 – 0.07 | 0.21 | 0.832 | 5035.00 |
| Valence | 0.15 | 0.12 – 0.17 | 10.20 | **<0.001** | 5035.00 | -0.24 | -0.26 – -0.21 | -17.12 | **<0.001** | 5035.00 | 0.20 | 0.17 – 0.23 | 13.56 | **<0.001** | 5035.00 | 0.21 | 0.18 – 0.25 | 13.65 | **<0.001** | 5035.00 |
| Arousal | 0.31 | 0.28 – 0.34 | 22.97 | **<0.001** | 5035.00 | 0.16 | 0.13 – 0.18 | 11.88 | **<0.001** | 5035.00 | 0.18 | 0.15 – 0.20 | 12.97 | **<0.001** | 5035.00 | 0.12 | 0.09 – 0.15 | 7.99 | **<0.001** | 5035.00 |
| Stress | 0.24 | 0.21 – 0.27 | 16.52 | **<0.001** | 5035.00 | 0.21 | 0.19 – 0.24 | 15.04 | **<0.001** | 5035.00 | -0.01 | -0.04 – 0.02 | -0.41 | 0.680 | 5035.00 | 0.16 | 0.13 – 0.19 | 10.02 | **<0.001** | 5035.00 |
| Environment1 | 0.03 | 0.01 – 0.06 | 2.61 | **0.009** | 5035.00 | -0.07 | -0.09 – -0.04 | -5.64 | **<0.001** | 5035.00 | 0.04 | 0.01 – 0.06 | 3.17 | **0.002** | 5035.00 | 0.03 | 0.00 – 0.06 | 2.17 | 0.030 | 5035.00 |
| DASS total | 0.07 | -0.00 – 0.15 | 1.94 | 0.053 | 5035.00 | 0.15 | 0.08 – 0.23 | 3.85 | **<0.001** | 5035.00 | 0.11 | 0.01 – 0.21 | 2.10 | 0.035 | 5035.00 | -0.04 | -0.12 – 0.05 | -0.87 | 0.384 | 5035.00 |
| Age | 0.12 | 0.06 – 0.18 | 3.92 | **<0.001** | 5035.00 | 0.04 | -0.03 – 0.10 | 1.13 | 0.259 | 5035.00 | -0.01 | -0.10 – 0.07 | -0.34 | 0.732 | 5035.00 | 0.08 | 0.01 – 0.14 | 2.21 | 0.027 | 5035.00 |
| Gender1 | -0.06 | -0.23 – 0.10 | -0.72 | 0.469 | 5035.00 | -0.14 | -0.32 – 0.03 | -1.60 | 0.110 | 5035.00 | 0.04 | -0.18 – 0.27 | 0.38 | 0.704 | 5035.00 | -0.02 | -0.20 – 0.16 | -0.20 | 0.844 | 5035.00 |

| | | | | | | | | | | | | | | | | | | | | |
|---|---|---|---|---|---|---|---|---|---|---|---|---|---|---|---|---|---|---|---|---|
| Gender2 | -0.08 | -0.27 – 0.11 | -0.85 | 0.395 | 5035.00 | 0.06 | -0.14 – 0.26 | 0.57 | 0.567 | 5035.00 | 0.17 | -0.09 – 0.43 | 1.26 | 0.210 | 5035.00 | -0.03 | -0.24 – 0.18 | -0.31 | 0.753 | 5035.00 |
| time | -0.03 | -0.04 – -0.02 | -4.41 | **<0.001** | 5035.00 | 0.00 | -0.01 – 0.01 | 0.12 | 0.902 | 5035.00 | 0.00 | -0.01 – 0.02 | 0.62 | 0.537 | 5035.00 | 0.02 | 0.01 – 0.03 | 2.85 | **0.004** | 5035.00 |
| Valence × Arousal | 0.00 | -0.02 – 0.02 | 0.17 | 0.868 | 5035.00 | -0.06 | -0.08 – -0.04 | -6.07 | **<0.001** | 5035.00 | 0.01 | -0.01 – 0.03 | 0.92 | 0.359 | 5035.00 | 0.04 | 0.01 – 0.06 | 3.05 | **0.002** | 5035.00 |
| TAS DIF × Valence | 0.01 | -0.02 – 0.04 | 0.59 | 0.557 | 5035.00 | -0.02 | -0.05 – 0.01 | -1.29 | 0.196 | 5035.00 | 0.02 | -0.01 – 0.06 | 1.23 | 0.220 | 5035.00 | 0.03 | -0.01 – 0.07 | 1.54 | 0.123 | 5035.00 |
| TAS DIF × Arousal | -0.02 | -0.05 – 0.01 | -1.39 | 0.166 | 5035.00 | 0.01 | -0.02 – 0.04 | 0.80 | 0.424 | 5035.00 | -0.01 | -0.04 – 0.03 | -0.36 | 0.721 | 5035.00 | 0.01 | -0.03 – 0.04 | 0.42 | 0.676 | 5035.00 |
| TAS DIF × Stress | 0.01 | -0.02 – 0.05 | 0.79 | 0.431 | 5035.00 | -0.00 | -0.04 – 0.03 | -0.14 | 0.886 | 5035.00 | -0.01 | -0.04 – 0.03 | -0.48 | 0.633 | 5035.00 | 0.01 | -0.02 – 0.05 | 0.78 | 0.434 | 5035.00 |
| TAS DDF × Valence | -0.03 | -0.07 – 0.00 | -1.82 | 0.069 | 5035.00 | 0.01 | -0.02 – 0.04 | 0.57 | 0.571 | 5035.00 | -0.00 | -0.04 – 0.03 | -0.15 | 0.882 | 5035.00 | -0.01 | -0.04 – 0.03 | -0.35 | 0.724 | 5035.00 |
| TAS DDF × Arousal | -0.01 | -0.04 – 0.03 | -0.32 | 0.751 | 5035.00 | -0.01 | -0.04 – 0.02 | -0.39 | 0.694 | 5035.00 | -0.01 | -0.04 – 0.02 | -0.78 | 0.433 | 5035.00 | -0.01 | -0.05 – 0.02 | -0.77 | 0.439 | 5035.00 |
| TAS DDF × Stress | -0.01 | -0.04 – 0.03 | -0.46 | 0.644 | 5035.00 | -0.02 | -0.05 – 0.02 | -1.07 | 0.283 | 5035.00 | 0.00 | -0.03 – 0.04 | 0.06 | 0.954 | 5035.00 | 0.02 | -0.02 – 0.05 | 0.84 | 0.403 | 5035.00 |
| TAS EOT × Valence | 0.01 | -0.01 – 0.04 | 0.91 | 0.364 | 5035.00 | 0.03 | 0.01 – 0.06 | 2.62 | **0.009** | 5035.00 | 0.03 | 0.01 – 0.06 | 2.46 | 0.014 | 5035.00 | -0.04 | -0.06 – -0.01 | -2.58 | **0.010** | 5035.00 |
| TAS EOT × Arousal | 0.00 | -0.02 – 0.03 | 0.18 | 0.858 | 5035.00 | -0.01 | -0.04 – 0.02 | -0.73 | 0.465 | 5035.00 | -0.01 | -0.04 – 0.02 | -0.83 | 0.408 | 5035.00 | 0.04 | 0.01 – 0.07 | 2.94 | **0.003** | 5035.00 |
| TAS EOT × Stress | 0.00 | -0.03 – 0.03 | 0.02 | 0.980 | 5035.00 | 0.01 | -0.01 – 0.04 | 0.97 | 0.330 | 5035.00 | 0.02 | -0.01 – 0.04 | 1.19 | 0.233 | 5035.00 | -0.05 | -0.08 – -0.02 | -3.23 | **0.001** | 5035.00 |
| (TAS DIF × Valence) × Arousal | 0.03 | 0.01 – 0.06 | 2.69 | **0.007** | 5035.00 | 0.01 | -0.02 – 0.03 | 0.49 | 0.625 | 5035.00 | 0.02 | -0.01 – 0.04 | 1.24 | 0.216 | 5035.00 | 0.00 | -0.03 – 0.03 | 0.05 | 0.959 | 5035.00 |

| | | | | | | | | | | | | | | | | | | | | |
|---|---|---|---|---|---|---|---|---|---|---|---|---|---|---|---|---|---|---|---|---|
| (TAS DDF × Valence) × Arousal | -0.01 | -0.04 – 0.01 | -0.93 | 0.354 | 5035.00 | -0.02 | -0.05 – 0.00 | -1.91 | 0.056 | 5035.00 | 0.01 | -0.02 – 0.03 | 0.70 | 0.482 | 5035.00 | -0.01 | -0.04 – 0.02 | -0.60 | 0.549 | 5035.00 |
| (TAS EOT × Valence) × Arousal | -0.02 | -0.04 – 0.00 | -1.57 | 0.117 | 5035.00 | 0.03 | 0.01 – 0.05 | 2.58 | **0.010** | 5035.00 | -0.01 | -0.03 – 0.01 | -0.75 | 0.455 | 5035.00 | 0.00 | -0.02 – 0.02 | 0.01 | 0.995 | 5035.00 |
| **Random Effects** | | | | | | | | | | | | | | | | | | | | |
| $\sigma^2$ | 0.58 | | | | | 0.54 | | | | | 0.59 | | | | | 0.71 | | | | |
| $\tau_{00}$ | 0.14 participant_code | | | | | 0.16 participant_code | | | | | 0.28 participant_code | | | | | 0.17 participant_code | | | | |
| N | 190 participant_code | | | | | 190 participant_code | | | | | 190 participant_code | | | | | 190 participant_code | | | | |
| Observations | 5063 | | | | | 5063 | | | | | 5063 | | | | | 5063 | | | | |
| Marginal $R^2$ / Conditional $R^2$ | 0.260 / 0.401 | | | | | 0.274 / 0.440 | | | | | 0.107 / 0.394 | | | | | 0.106 / 0.278 | | | | |

**Supplementary Table 19.** Additional analysis controlling for time (probe position). LMM summary tables for the interaction effects of TAS-20 total and Social Environment on 4 thought dimensions

| | **Future-self focus** | | | | | **Intrusive distraction** | | | | | **Sensory engagement** | | | | | **Task-focus** | | | | |
|---|---|---|---|---|---|---|---|---|---|---|---|---|---|---|---|---|---|---|---|---|
| *Predictors* | *Estimates* | *CI* | *Statistic* | *p* | *df* | *Estimates* | *CI* | *Statistic* | *p* | *df* | *Estimates* | *CI* | *Statistic* | *p* | *df* | *Estimates* | *CI* | *Statistic* | *p* | *df* |
| (Intercept) | 0.06 | -0.15 – 0.27 | 0.55 | 0.581 | 5052.00 | 0.13 | -0.09 – 0.35 | 1.17 | 0.242 | 5052.00 | -0.14 | -0.39 – 0.11 | -1.12 | 0.264 | 5052.00 | -0.11 | -0.32 – 0.10 | -1.02 | 0.307 | 5052.00 |
| TAS total | -0.12 | -0.21 – -0.03 | -2.54 | **0.011** | 5052.00 | -0.08 | -0.18 – 0.01 | -1.69 | 0.091 | 5052.00 | -0.01 | -0.12 – 0.10 | -0.19 | 0.847 | 5052.00 | -0.00 | -0.09 – 0.09 | -0.04 | 0.971 | 5052.00 |
| Environment1 | -0.09 | -0.12 – -0.07 | -6.97 | **<0.001** | 5052.00 | 0.07 | 0.04 – 0.10 | 5.34 | **<0.001** | 5052.00 | -0.08 | -0.10 – -0.05 | -6.25 | **<0.001** | 5052.00 | -0.08 | -0.10 – -0.05 | -5.63 | **<0.001** | 5052.00 |
| DASS total | 0.14 | 0.06 – 0.23 | 3.19 | **0.001** | 5052.00 | 0.29 | 0.20 – 0.38 | 6.12 | **<0.001** | 5052.00 | 0.10 | -0.01 – 0.20 | 1.75 | 0.080 | 5052.00 | -0.02 | -0.10 – 0.07 | -0.36 | 0.719 | 5052.00 |
| Age | 0.16 | 0.09 – 0.24 | 4.19 | **<0.001** | 5052.00 | 0.04 | -0.03 – 0.12 | 1.11 | 0.267 | 5052.00 | 0.01 | -0.08 – 0.10 | 0.17 | 0.864 | 5052.00 | 0.10 | 0.03 – 0.18 | 2.71 | **0.007** | 5052.00 |
| Gender1 | -0.02 | -0.23 – 0.19 | -0.16 | 0.870 | 5052.00 | -0.14 | -0.35 – 0.08 | -1.24 | 0.216 | 5052.00 | 0.08 | -0.17 – 0.33 | 0.60 | 0.550 | 5052.00 | 0.00 | -0.21 – 0.21 | 0.00 | 0.997 | 5052.00 |
| Gender2 | 0.07 | -0.16 – 0.30 | 0.59 | 0.554 | 5052.00 | 0.12 | -0.12 – 0.36 | 0.99 | 0.322 | 5052.00 | 0.20 | -0.08 – 0.48 | 1.40 | 0.161 | 5052.00 | -0.00 | -0.23 – 0.23 | -0.03 | 0.973 | 5052.00 |
| time | -0.02 | -0.03 – -0.01 | -2.76 | **0.006** | 5052.00 | -0.00 | -0.02 – 0.01 | -0.31 | 0.754 | 5052.00 | 0.01 | 0.00 – 0.03 | 2.07 | 0.039 | 5052.00 | 0.03 | 0.01 – 0.04 | 3.48 | **<0.001** | 5052.00 |
| TAS total × Environment1 | -0.00 | -0.03 – 0.03 | -0.03 | 0.979 | 5052.00 | 0.01 | -0.02 – 0.03 | 0.55 | 0.580 | 5052.00 | -0.03 | -0.06 – -0.01 | -2.67 | **0.008** | 5052.00 | -0.01 | -0.04 – 0.02 | -0.64 | 0.524 | 5052.00 |
| **Random Effects** | | | | | | | | | | | | | | | | | | | | |
| $\sigma^2$ | 0.72 | | | | | 0.69 | | | | | 0.65 | | | | | 0.76 | | | | |
| $\tau_{00}$ | 0.23 participant_code | | | | | 0.25 participant_code | | | | | 0.34 participant_code | | | | | 0.22 participant_code | | | | |
| N | 190 participant_code | | | | | 190 participant_code | | | | | 190 participant_code | | | | | 190 participant_code | | | | |

| | | | | |
|---|---|---|---|---|
| Observations | 5063 | 5063 | 5063 | 5063 |
| Marginal $R^2$ / Conditional $R^2$ | 0.058 / 0.287 | 0.072 / 0.318 | 0.019 / 0.360 | 0.019 / 0.242 |
